# A Semi-Automated Method to Extract Green and Non-Photosynthetic Vegetation Cover from RGB Images in Mixed Grasslands

**DOI:** 10.3390/s20236870

**Published:** 2020-12-01

**Authors:** Dandan Xu, Yihan Pu, Xulin Guo

**Affiliations:** 1 Department of Ecology, College of Biology and the Environment, Nanjing Forestry University, Nanjing 210037, China; pyh1997@njfu.edu.cn; 2 Co-Innovation Center for Sustainable Forestry in Southern China, Nanjing Forestry University, Nanjing 210037, China; 3 Department of Geography and Planning, University of Saskatchewan, 117 Science Place, Saskatoon, SK S7N5C8, Canada; xulin.guo@usask.ca

**Keywords:** CAI, green cover, NDVI, RGB imagery, standing dead matter

## Abstract

Green (GV) and non-photosynthetic vegetation (NPV) cover are both important biophysical parameters for grassland research. The current methodology for cover estimation, including subjective visual estimation and digital image analysis, requires human intervention, lacks automation, batch processing capabilities and extraction accuracy. Therefore, this study proposed to develop a method to quantify both GV and standing dead matter (SDM) fraction cover from field-taken digital RGB images with semi-automated batch processing capabilities (i.e., written as a python script) for mixed grasslands with more complex background information including litter, moss, lichen, rocks and soil. The results show that the GV cover extracted by the method developed in this study is superior to that by subjective visual estimation based on the linear relation with normalized vegetation index (NDVI) calculated from field measured hyper-spectra (R^2^ = 0.846, *p* < 0.001 for GV cover estimated from RGB images; R^2^ = 0.711, *p* < 0.001 for subjective visual estimated GV cover). The results also show that the developed method has great potential to estimate SDM cover with limited effects of light colored understory components including litter, soil crust and bare soil. In addition, the results of this study indicate that subjective visual estimation tends to estimate higher cover for both GV and SDM compared to that estimated from RGB images.

## 1. Introduction

Fractional vegetation cover, defined as the percentage of vegetation that is vertically projected in a unit area [1], is an important indicator of plant growth [2], vegetation status [3], crop health [4], habitat selection [5] and ecosystem change [6]. Vegetation cover is also closely related to leaf area index, net primary productivity, biomass, soil stability, photosynthesis and ecological processes [6,7,8]. In grassland ecosystems, systematic, accurate and repeatable surveys of vegetation cover are essential for monitoring grassland condition, protection of soil erosion and grassland management [9,10,11].

Fractional cover estimation in grasslands relies on field measurements combined with remote sensing technology [7]. Field measured cover data are fundamental for quantitative models using remotely sensed images [12,13] and are necessary to validate empirical models to estimate grassland cover [6,14]. Visual (i.e., non-destructive) estimation is a commonly used field method for grassland vegetation cover [15]. This is a rapid and repeatable evaluation of vegetation cover [16] and is sufficiently accurate for relative (as opposed to absolute) assessments of cover data [7]. However, visual estimation is subjective and prone to observer biases [5], which can lead to inconsistent data among observers and observation periods [3,16]. Attempts have been made to reduce visual estimation bias in grassland vegetation cover, including fishnet grids [9], cardboard cutouts of specific shapes and size, and observer training [5]. However, trained observers are not able to distinguish cover intervals or changes less than 10% [16].

An alternative approach for field vegetation cover measurement is analysis of low altitude RGB (true color composition: Red, Green, Blue) images taken with digital cameras in the field [7]. Because field-taken digital RGB images with high spatial resolution potentially provide more accurate estimation of vegetation cover than visual methods by reducing the impact of human subjectivity [1,14]. They have been widely used for estimating forest canopy cover i.e., gap fraction analysis, [17,18], forest understory cover [16], crop cover [4,19,20,21,22], crop residual cover [23] and grassland vegetation cover [5,24]. 

Many studies have demonstrated the potential of field-taken digital RGB images to extract the green vegetation (GV) coverage [10,24,25,26] or crop residuals from soil background [23]. However, there has been less success separating GV and non-photosynthetic vegetation (NPV) using the RGB images. In arid grasslands, green and senescent vegetation, important indicators of grassland managements, are often intermixed and very difficult to differentiate [27]. The situation is even more complex in mixed grasslands with more components, including standing dead matter (SDM), litter, soil crust (moss and lichen), rocks and bare soil, in a heterogeneous mix [13].

The heterogeneity of mixed grassland components is challenging not only for GV cover estimation but also for SDM extraction from digital RGB images. Current analytical methods using digital RGB images, include unsupervised classification, supervised classification with training sites, objected oriented classification, RGB-based color indices and threshold algorithms [1,2,5,6,9,12,14,15,27]. Nearly all methods require human intervention (e.g., the SamplePoint software requires user inputs of classification for each sample point [28]), lack automation, batch processing capabilities and extraction accuracy [1,3,18,29]. Therefore, there is an opportunity to develop a fast, objective, repeatable and consistent analytical method to improve mixed grassland cover estimation, which would effectively support fieldwork for collecting low-altitude cover data. We aimed to develop a digital image analysis method to extract both GV and SDM fraction cover from field-taken digital RGB images with semi-automated batch processing capabilities. Our specific objectives were to: (1) extract GV and SDM cover separately from field-taken RGB images semi-automatically; (2) validate the extracted GV and SDM cover using hyperspectral vegetation indices. 

## 2. Materials and Methods

### 2.1. Study Area

**Figure 1:**
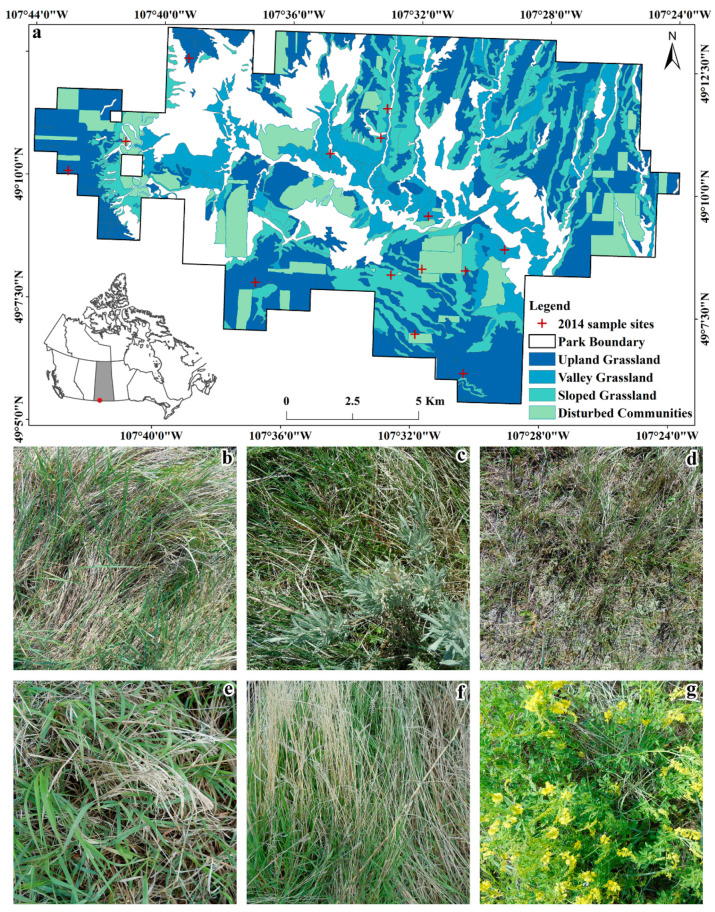
Vegetation communities in Grassland National Park (GNP). (**a**) Vegetation communities in GNP first surveyed in 1983 and disturbed community data updated in 1995. (**b**) Upland grassland. (**c**) Valley grassland. (**d**) Sloped grassland. (**e**) Disturbed community with Smooth Brome (*Bromus inermis Layss.*). (**f**) Disturbed community with Crested Wheatgrass (*Agropyron cristatum*). (**g**) Disturbed community with Sweet Clover (*Melilotus officinalis*).

This research is conducted in Grasslands National Park (GNP: West Block, 49° N, 107° W, Figure 1) in the southern part of Saskatchewan, Canada. The study area is characterized as a semi-arid mixed prairie ecosystem (i.e., annual precipitation: 340 mm; annual mean temperature: 3.4 °C) [13]. Three main vegetation communities are upland (Figure 1b), sloped (Figure 1d) and valley (Figure 1c) grasslands, including disturbed herbaceous communities (Figure 1e–g). The dominant species are described in Table 1. In 1984, GNP was first acquired as a national park [30], at which time all larger grazers were removed until 2006. This has led to approximately 30 years of accumulation of a large amount NPV including SDM and litter, which brings a challenge to estimate GV and SDM cover using field collected digital RGB images. 

### 2.2. Field Data Collection

Fieldwork was performed during 20 June to 2 July 2014 in the peak growing season of GNP. A stratified random sampling design was used to select 14 sites with consideration of accessibility (Figure 1: 4 sites in upland grassland, 5 in sloped grassland, 3 in valley grassland and 2 in disturbed communities). Two 100 m transects were surveyed perpendicular to one another and crossing in the center at each site. Twenty, 50 cm × 50 cm quadrats at 10 m intervals (excluding the center point) were surveyed along the transects. 

This design is intended to collect the heterogeneity of biophysical parameters on the representative grasslands. Percent ground cover, including grass, shrub, forb, SDM, litter, moss, lichen, rock and bare soil coverage were visual estimated at each quadrat. The descriptive statistics of GV cover (i.e., sum of grass and forb cover), SDM and NPV cover (i.e., sum of SDM and litter cover) are shown in Table 2. Nadir (i.e., downward facing) RGB images were taken by a commercially available digital camera (Nikon S8000, Nikon Imaging Japan Inc., Tokyo, Japan) at each quadrat (i.e., the corresponding RGB pictures for each quadrat in the 14 sites of Table 2 are listed in Supplementary S2) at 1 m above the ground. A 0° camera angle enables fractional cover estimation when compared to oblique angles tested [31]. Hyper-spectra (wavelength from 350 nm to 2500 nm) were also measured at each quadrat with an Analytical Spectral Devices (ASD) field-portable FieldSpec^®^ Pro Spectroradiometer between 10:00 a.m. and 14:00 p.m. under clear sky (i.e., without any cloud cover). 

### 2.3. Methods

**Figure 2:**
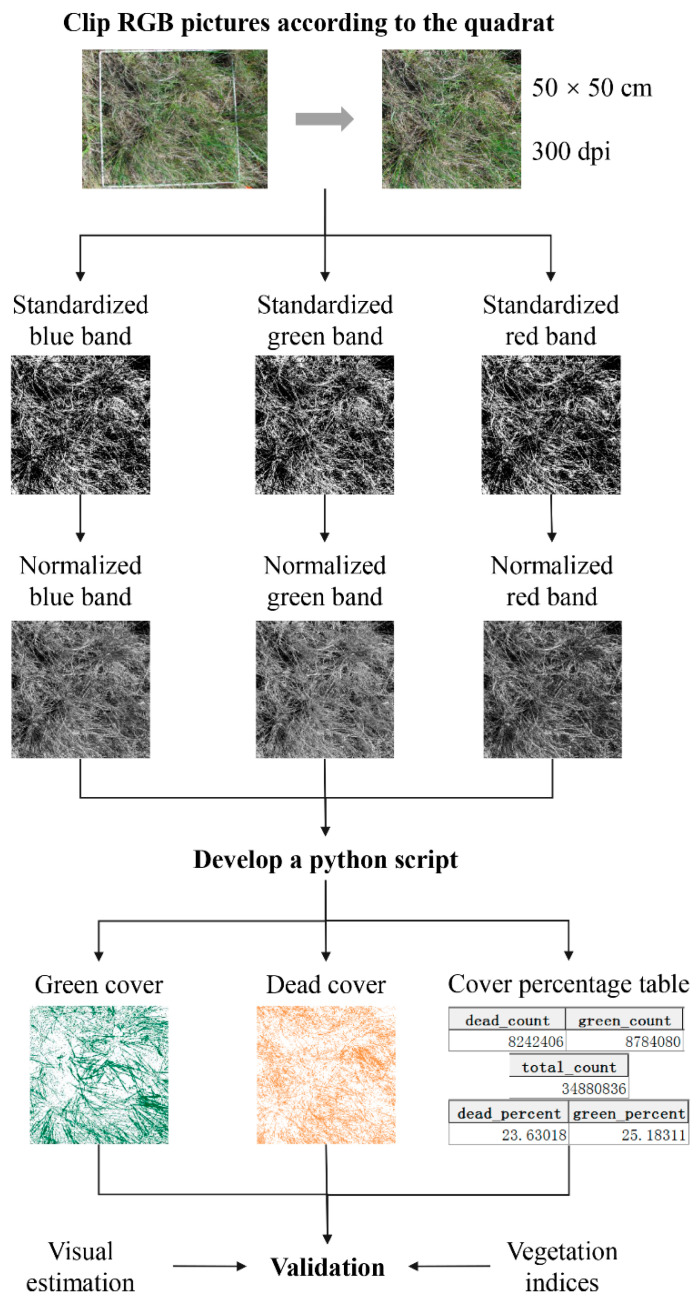
Flowchart of the methodology for this study.

The methodological workflow for the proposed semi-automatic method included preprocessing digital RGB images, developing a python script to extract GV and SDM separately and calculating GV and SDM percentage cover automatically (Figure 2). The result of semi-automatically estimated GV and SDM cover with visual estimated cover data and vegetation indices were validated based on hyperspectral remote sensing (Figure 2).

#### 2.3.1. Pre-Processing for the Field-Taken RGB Images

RGB images were first cropped to the quadrat area and then processed to their actual size (50 cm × 50 cm) with 300 pixels/inch (dpi) using Adobe Photoshop CS6 (Figure 2). 

Because light conditions differed slightly among field-taken RGB images, blue, green and red bands of cropped pictures were standardized independently to maintain consistency among study sites (Equation (1)).

(1) DNstd=(DN−DN¯)std(DN)

where $DN_{std}$ is the standardized pixel value, DN is the original pixel value, $\bar{DN}$ is the mean value of all the pixel values in a single band, and std(x) is the standard deviation for all the pixel values in a single band.

After each band for all the pictures was standardized, all the pixel values fit in a range from −1 to 1. Standardized images were then normalized (Equation (2)) as images with pixel value range from 0 to 1023 (10 bit integer data format).

(2) DNnor=1023×(DNstd−min(DNstd))(max(DNstd)−min(xDNstd))

where $DN_{nor}$ is the normalized pixel value, $DN_{std}$ is standardized pixel value, $\min\left(xDN_{std}\right)$ is the minimum value of all the pixel values in each standardized band, and $\max\left(DN_{std}\right)$ is the maximum of all the pixel values in each standardized band.

#### 2.3.2. Developing a Python Script to Semi-Automate GV and SDM Cover Extraction from Preprocessed RGB Images

GV pixels in the RGB images were extracted based on the spectral characteristics of GV (i.e., reflectance for green leaves in the green band is larger than that in both red and blue bands; Equation (3)). GV pixels were masked out before further process for extracting SDM pixels.

(3) $$\left(DN_{nor}^{G}-DN_{nor}^{R}\right)>g_{1}\;\mathrm{and}\;\left(DN_{nor}^{G}-DN_{nor}^{B}\right)>g_{2}$$

where $DN_{nor}^{G}$, $DN_{nor}^{R}$, $DN_{nor}^{B}$ are the normalized pixel value for the green band, red band and blue band of the field-taken RGB pictures, $g_{1},\;g_{2}$ are constants (i.e., default values were set to 60 in this study). The values of $g_{1},\;g_{2}$ were determined after exploring the spectral characteristics of green leaves for narrow leaved native grasses, shrubs, invasive species in disturbed communities (their values are discussed in Section 3.1).

Even though SDM has similar spectral characteristics such as litter, soil crust and bare soil, SDM in the canopy have much brighter color tong in all the three visible bands in field-taken RGB images because the understory components receive limited sunlight in comparison with the vegetation canopy. Therefore, SDM is extracted based on the criteria that the SDM has higher pixel values than the understory components in the normalized visible bands after GV pixels were removed (Equation (4)).

(4) $$DN_{nor}^{R}>d\times \bar{DN_{nor}^{R}}\;\mathrm{and}\;DN_{nor}^{G}>d\times \bar{DN_{nor}^{G}}\;\mathrm{and}\;DN_{nor}^{B}>d\times \bar{DN_{nor}^{B}}$$

where $DN_{nor}^{G}$, $DN_{nor}^{R}$, $DN_{nor}^{B}$ are the normalized pixel values for green, red and blue bands of the field-taken RGB images, $\bar{DN_{nor}^{G}}$, $\bar{DN_{nor}^{R}}$, $\bar{DN_{nor}^{B}}$ are the mean pixel values of the green, red and blue normalized bands of the field-taken RGB images, d is a constant set as a default value of 1 for our study. The setting of constant d was discussed in Section 3.2 when litter and soil background with light color tong challenging the extraction of SDM.

After GV and SDM pixels were separated from the pre-processed RGB images, GV and SDM cover were calculated. Both GV and SDM pixel counts were divided by total pixel counts for fractional cover. These cover estimations were compared with field observed cover data by visual estimation. 

All classification and cover estimation processes for both GV and SDM were conducted using a python script developed in this study (see the stand-alone python script and ArcToolbox in the Supplementary Materials and the description of ArcToolbox in Supplementary S1). 

#### 2.3.3. Validation of Extracted GV and SDM Cover from RGB Images

Alternative methods for GV and NPV cover estimation (i.e., vegetation indices based on hyperspectral remote sensing) were used to validate estimated GV and SDM cover from field-taken digital RGB images. Normalized difference vegetation index (NDVI), an index strongly correlated to GV, has been widely used to evaluate GV cover in grasslands [10]. The cellulose absorption index (CAI) is effective for estimating NPV fractional cover (i.e., including SDM and litter cover) from GV and soil background [32,33,34]. Therefore, NDVI calculated from field measured hyper-spectra (Equation (5)) was used to test the accuracy of GV cover extracted from field-taken RGB images based on linear regression analysis in R software (i.e., it is written by John Chambers and his colleagues of the Bell Laboratories, Murray Hill, NJ, USA). The CAI calculated from field-collected hyper-spectra (Equation (6)) was used to validate the accuracy of SDM extraction from field-taken RGB images with linear regression analysis in R software (i.e., the NPV cover used for linear regression with CAI is the sum of RGB image extracted SDM cover and visual estimated litter cover).

(5) NDVI =(ρ800−ρ670)(ρ800+ρ670)

where $\rho_{800}$ and $\rho_{670}$ are the reflectance in the wavelength of 800 nm and 670 nm from field-collected hyper-spectra.

(6) $$CAI\;=100\left(0.5\left(\rho_{2030}+\rho_{2210}\right)-\rho_{2100}\right)$$

where $\rho_{2030}$, $\rho_{2100}$ and $\rho_{2210}$ are the reflectance in the wavelength of 2030 nm, 2100 nm and 2210 nm from field-collected hyper-spectra. 

## 3. Results

### 3.1. Determination of the Constants $g_{1},g_{2}$ for GV Extraction

After data exploration of 25 sample RGB images for different species in different conditions (Table 3, see the sample RGB photos in Supplementary S3), values of $g_{1},\;g_{2}$ were found to exceed 60 when the photograph was taken without high exposure under strong light (normal light conditions). Under normal light conditions, the minimum values of $g_{1},\;g_{2}$ for broad-leaved vegetation (e.g., Sweet Clover) was higher than narrow-leaved grasses (Table 3: id 5 and 6). When the RGB images were taken with high exposure or when the vegetation had begun to senesce, constant $g_{1}$ needed to be set at a value lower than 60 (32.09–44.13; Table 3). The value of $g_{2}$ was not affected by these conditions but was affected by bluish leaves (i.e., dominated western wheat grass and sagebrush). In these instances, the value of $g_{2}$ needed to be set lower (i.e., 32.09–40.12; Table 3). 

### 3.2. Setting of the Constant d for SDM Extraction

**Figure 3:**
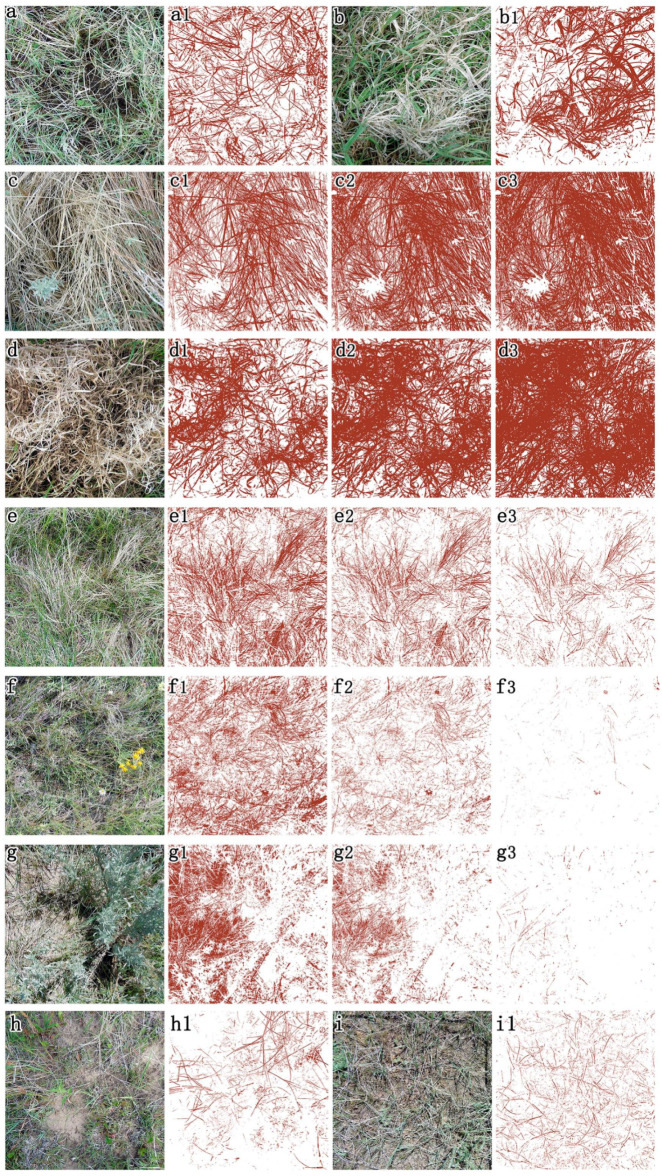
Extracted SDM from field-taken RGB images. (**a**) RGB image taken in valley grassland. (**a1**) extraction of standing dead matter (SDM) from (**a**) with d = 1. (**b**) RGB image taken in disturbed communities. (**b1**) Extraction of standing dead material from (b) with d = 1. (**c**) RGB image taken in valley grassland. (**c1**–**c3**) Extraction of SDM from (**c**) with d = 1, 0.7, 0.5, respectively. (**d**) RGB image taken in sloped grassland. (**d1**–**d3**) Extraction of SDM from (**d**) with d = 1, 0.7, 0.5, respectively. (**e**) RGB image taken in sloped grassland. (**e1**–**e3**) Extraction of SDM from (**e**) with d = 1, 1.2, 1.5, respectively. (**f**) RGB image taken in upland grassland. (**f1**–**f3**) Extraction of SDM from (**f**) with d = 1, 1.5, 2, respectively. (**g**) RGB image taken in valley grassland. (**g1**–**g3**) Extraction of SDM from (**g**) with d = 1, 1.5, 2, respectively. (**h**) RGB image taken in disturbed communities. (**h1**) Extraction of SDM from (**h**) with d = 1.5. (**i**) RGB image taken in valley grassland. (**i1**) Extraction of SDM from (**i**) with d = 1.7.

SDM, as one part of grassland canopy, has higher pixel values in all three bands of field-taken RGB images than bare soil, litter and soil crust after GV pixels were masked out (Equation (4)). Therefore, it is effectively extracted by default constant d (set at 1) when the understory background, including soil, litter and soil crust, is dark (Figure 3a,a1,b,b1). When the percentage of the dead component in the canopy is high (i.e., visual estimation of dead material for Figure 3c is 87% and for Figure 3d is 90%), SDM in the canopy is brighter than the lower layer (Figure 3c,d), including litter that has similar spectral characteristics with SDM. In this situation, d must be set lower to extract more standing dead material in the darker, lower canopy (Figure 3c,d). The extracted fraction of SDM is 41.7% (Figure 3c1), 61.5% (Figure 3c2) and 72.1% (Figure 3c3) when d was set as 1, 0.7 and 0.5, respectively (Figure 3c) and the estimated percentage of SDM was 43.7% (Figure 3d1), 65.6% (Figure 3d2) and 80.3% (Figure 3d3) when d was set as 1, 0.7 and 0.5, respectively (Figure 3d). 

Light colored, undecomposed litter has a large effect on the extraction of standing dead material (Figure 3e: visual estimation for standing dead materials is 40% and for litter is 20%) when the canopy cover (i.e., sum of GV and SDM cover) is low, thus, d needs to be set higher than the default value of one. The extracted SDM was 30.8% (Figure 3e1), 20.2% (Figure 3e2) and 6.5% (Figure 3e3) when d was set at 1, 1.2 and 1.5, respectively. Light colored soil crust as the canopy background also has a strong influence in SDM extraction in the study area (Figure 3f: visual estimation for standing dead materials is 15%). In this case, d must be set higher to reduce the influence of soil crust (i.e., moss and lichen). The extracted SDM was 27.8% (Figure 3f1), 8.6% (Figure 3f2) and 0.4% (Figure 3f3) when d was set at 1, 1.5 and 2, respectively. Light colored bare soil also influences the extraction of SDM with a default value of d (Figure 3g: visual estimation for standing dead material is 10%). d must be set higher to reduce the effects of bare soil (Figure 3g3: d was set at 2) providing high accuracy of standing dead material extraction (0.8%, Figure 3g3) compared to 11.9% extraction where some bare soil pixels were extracted (Figure 3g2: d was set at 1.5) and 25.9% extraction where a large amount of bare soil pixels were present in the extraction results (Figure 3g1: d was set at 1). Especially when canopy cover is low, light colored bare soil still has great effects on SDM extraction even though d is set appropriately for extracting SDM pixels (Figure 3h1: d was set at 1.5; Figure 3i1: d was set at 1.7).

### 3.3. GV and SDM Cover Estimated from RGB Images

**Figure 4:**
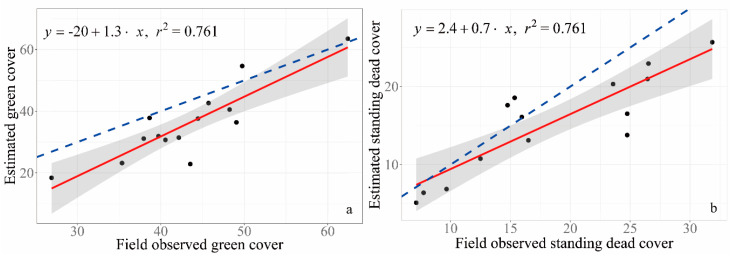
Comparing estimated green and dead cover with field observed cover (the red solid line is the regression line; the blue line is 1:1). (**a**) Comparison between green vegetation (GV) cover from visual estimation (i.e., field observed green cover) and that from GV extraction by the method developed in this study. (**b**) Comparison between SDM from visual estimation (i.e., field observed standing dead cover) and that from SDM extraction from the method of this study.

Compared to subjective visual estimation, GV cover is under-estimated by the method developed in this study (Figure 4a). The difference between estimated GV cover and subjective visual estimated GV cover is larger when GV cover is lower (Figure 4a). Estimated cover of SDM in this study is under-estimated compared to that from subjective visual estimation. The underestimation becomes more distinct when SDM cover is higher (Figure 4b). 

### 3.4. Validation of GV and NPV Estimated from RGB Images

**Figure 5:**
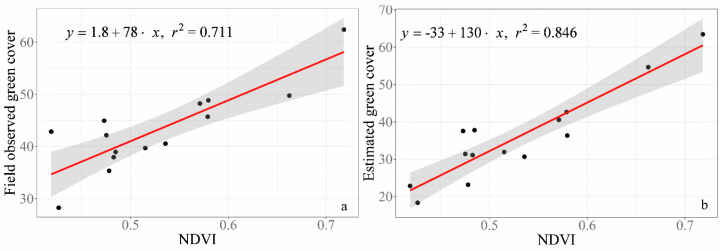
Validation of RGB extracted green cover. (**a**) The relationship between normalized vegetation index (NDVI) and visual estimated GV cover (i.e., field observed green cover). (**b**) The relationship between NDVI and GV cover extracted by the method of this study.

Based on the relationship between GV cover and NDVI, the estimated GV cover (Figure 5b: R^2^ = 0.846, *p* < 0.001) in this study is more precise than that from subjective visual estimation (Figure 5a: R^2^ = 0.711, *p* < 0.001). 

**Figure 6:**
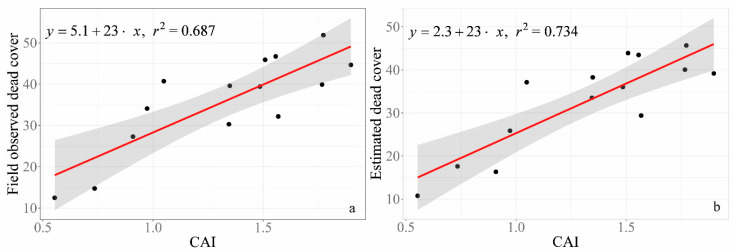
Validation of RGB extracted standing dead cover. (**a**) The relationship between cellulose absorption index (CAI) and visual estimated non-photosynthetic vegetation (NPV) cover (i.e., sum of visual estimated SDM and litter cover). (**b**) The relationship between CAI and the estimated NPV cover (i.e., sum of extracted cover of standing dead matter (SDM) by the method of this study and visually estimated cover of litter in the field).

Theoretically, CAI can be used to evaluate NPV cover including cover of SDM and litter. There is no significant linear relationship of CAI and estimated SDM cover. Therefore, cover used for validation is comprised of total NPV including SDM and litter, thus, the estimated dead cover (Figure 6b) is the sum of the estimated cover of SDM and field observed litter cover. The R square of estimated dead cover increased from 0.687 (*p* < 0.001) with subjective visual estimation to 0.734 (*p* < 0.001) with the linear regression of CAI (Figure 6a).

## 4. Discussion

### 4.1. GV and SDM Cover Estimation Based on RGB Images and Visual Estimation

Our semi-automated method to classify RGB images predicts lower GV cover than visual estimates (Figure 4a). The difference between visual estimation and extraction of GV coverage from RGB images is higher when the GV coverage is relatively low (Figure 4a). Previous research also suggests that subjective visual estimation tends to predict higher cover (i.e., overestimation) than GV cover estimates from digital image analysis based on field-taken RGB pictures [8,35]. Macfarlane and Ogden’s results show that subjective visual estimation accuracy is ±10–20% [16]. This indicates that green cover collected by visual estimation may consistently overestimate real GV cover. Moreover, the linear regression of GV cover and NDVI, an alternative method based on remote sensing imagery for GV cover estimation, indicates that GV cover extracted from field-taken RGB images (Figure 5b: R^2^ = 0.846, *p* < 0.001) in this study is superior to the GV cover from the subjective visual estimation in the field (Figure 5a: R^2^ = 0.711, *p* < 0.001). We also compared our GV extraction with the extraction results from Canopeo (http://www.canopeoapp.com), a powerful tool for measuring GV cover in grassland [36] which has been proven to show good performance for measuring GV cover of narrow-leaved vegetation [11]. The comparison results show that the extracted GV cover with our semi-automated method is consistent with that of Canopeo (R^2^ = 0.86, *p* < 0.001) and GV cover by Canopeo also has high linear relationship with NDVI (R^2^ = 0.85, *p* < 0.001). It indicates that the method developed in this study has high potential to assess GV cover effectively and accurately. Moreover, this method has batch capacity, which would effectively support field data collection. 

The results of linear regression between NPV cover and CAI (an alternative method for NPV cover estimation based on remote sensing approaches) show that cover estimates from RGB images in this study (Figure 6b: R^2^ = 0.734, *p* < 0.001) are superior to subjective visual estimates (Figure 6a: R^2^ = 0.687, *p* < 0.001). Estimated cover of SDM based on field-taken RGB images in this study is lower than subjective visual estimates. The difference between visual estimation and extraction from RGB pictures for SDM cover becomes larger when SDM cover increases. When SDM cover is larger, SDM in the lower layer is darker than in the top layer, which challenges the extraction of SDM from field-taken RGB images. Therefore, SDM cover might be underestimated by RGB images when the cover of SDM is very high. However, SDM cover might be overestimated from RGB images when SDM cover is low with background soil that has a light tone. 

### 4.2. Estimated Green Cover from RGB Pictures

**Figure 7:**
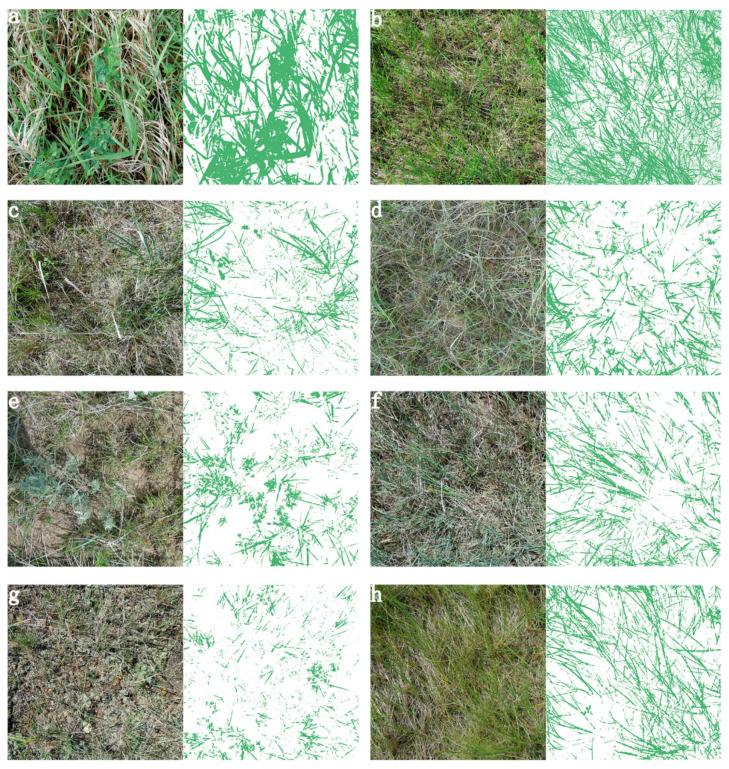
Extracted green vegetation from RGB images. (**a**) RGB image taken in disturbed communities; extraction of green vegetation (GV) with $g_{1}$ = 60 and $g_{2}$ = 60. (**b**) RGB image taken in upland grassland; extraction of GV with $g_{1}$ = 60 and $g_{2}$ = 60. (**c**) RGB image taken in valley grassland; extraction of GV with $g_{1}$ = 60 and $g_{2}$ = 60. (**d**) RGB image taken in sloped grassland; extraction of GV with $g_{1}$ = 60 and $g_{2}$ = 60. (**e,f**) RGB image taken in valley grassland; extraction of GV with $g_{1}$ = 40 and $g_{2}$ = 32. (**g**) RGB image taken in sloped grassland; extraction of GV with $g_{1}$ = 60 and $g_{2}$ = 40. (**h**) RGB image taken in upland grassland; extraction of GV with $g_{1}$ = 30 and $g_{2}$ = 60.

After standardizing and normalizing red (band 1), green (band 2) and blue (band 3) from the RGB images (Equations (1) and (2)), we found that the green band had the highest pixel value for green vegetation. Therefore, the constants $g_{1},\;g_{2}$ (Equation (3)) can be used as thresholds to separate green vegetation from SDM, litter, soil crust (moss and lichen), rocks and bare soil. In this study, green vegetation was extracted accurately in most cases when $g_{1},\;g_{2}$ were set to the default value of 60 (Figure 7a–d). 

However, default values of $g_{1},\;g_{2}$ should be tied to vegetation type, phenology stage, soil crust and the light conditions when taking RGB pictures. Previous research indicates that GV cover extracted from field-taken RGB images is influenced by resolution, exposure and ground complexity [3]. In our study area, sagebrush and western wheatgrass are a pale blue color (Figure 7e,f). For this case, we adjusted $g_{2}$ lower (32) and moved $g_{1}$ to be lower than 60 to capture more sage leaves (Figure 7e,f; $g_{1},\;g_{2}$ were set as 40 and 32, respectively). When soil crust, especially moss with green color, influences green vegetation extraction from RGB pictures, $g_{1}$ could be set higher than $g_{2}$ because the difference between normalized green band and normalized blue band is far greater than the difference between normalized green band and normalized red band for green moss (Figure 7g; $g_{1},\;g_{2}$ were set as 60 and 40, respectively). Because the python script is designed to extract GV and SDM pixels separately to quantify ground cover of GV and SDM (Figure 7h; $g_{1},\;g_{2}$ were set as 30 and 60, respectively). If the vegetation is in early or late senescence, $g_{1}$ should be set as a lower value than the default 60 to extract more GV pixels which are not completely senesced. 

When RGB photographs were taken near noon, the issue of high exposure reduced the greenness in the green band. Therefore, $g_{1}$ should be set lower than 60 (32, Table 3). Even though it improves the accuracy to extract green vegetation from RGB images by changing the parameters $g_{1},\;g_{2}$ in the python script we developed, the effects of sage and green moss are still not eliminated. They are, however, reduced. Green vegetation is effectively extracted by our method when GV was overlapping in the original RGB images (Figure 7) and compared with the GV extraction from Canopeo. When green moss was present, this principle did not necessarily hold (Figure 7g). For all these limitations of GV cover estimation, we suggest taking RGB pictures of each quadrat in the maximum growing season to avoid the senesced vegetation issue and avoid high exposure issue at noon. 

### 4.3. Estimated SDM from RGB Images

SDM has high pixel values in all three normalized visible bands. Equation (4) was designed based on this concept. Green vegetation was masked out for the normalized RGB images before extracting SDM to completely eliminate effects from green canopy, but the averaged pixel values of each normalized band were calculated before the green cover was masked out (Equation (4)). The parameter d is designed to separate standing dead cover from litter and a light soil background (Equation (4)). In this way, SDM was extracted accurately under moderate and high canopy cover (Figure 3a,b; d was set as default value 1). 

Undecomposed litter has similar spectral characteristics as SDM. When the canopy cover (sum of GV and SDM cover) is low, litter has large effects on the accuracy for extracting SDM pixels. In normalized RGB images, litter is slightly darker than SDM in all the three bands because light exposure differs for the canopy and understory, and the color tone of litter becomes darker when it begins to decompose. To reduce the impact of litter when extracting SDM, the constant d was set higher (in the range one to two in this study) than the default value of one (Figure 3e3; d was set as 1.5). 

**Figure 8:**
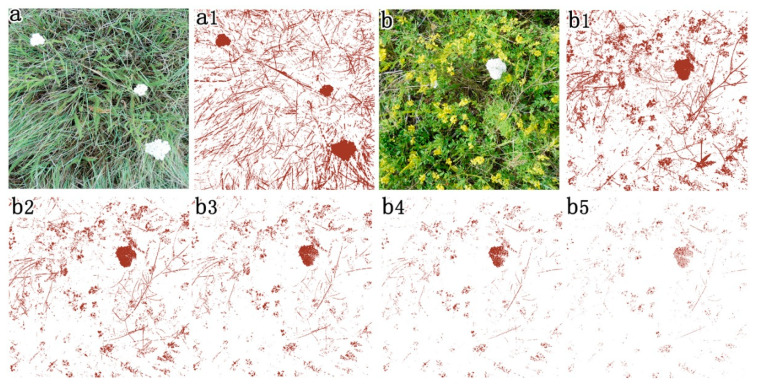
Flower effects on the extraction of standing dead matter (SDM) from RGB images. (**a**) RGB image taken in valley grassland. (**a1**) Extraction of SDM from (**a**) with d = 1. (**b**) RGB image taken in disturbed communities. (**b1**–**b5**) Extraction of standing dead materials from (**b**) with d = 1, 1.5, 1.7, 1.8 and 1.9, respectively.

Dry bare soil with light color tone is another issue for extracting SDM when the canopy cover is low. The errors caused by light soil background can be reduced by setting the constant d to a higher value (Figure 3g3; d was set as two). When the actual SDM cover is high, SDM cover may be underestimated (Figure 3c,d) with our method. SDM in the lower canopy has a darker color tone than that in the upper canopy when the SDM cover is high. Thus, the SDM in the lower canopy will be treated as litter to be excluded in the output of SDM cover (Figure 3c1,d1). In this specific case, we set the parameter d lower than the default value 1 (Figure 3c1,d1 when d was set as default value 1; Figure 3c2,d2 when d was set as 0.7; Figure 3c3,d3 when d was set as 0.5). In addition, the extracted results of SDM were overestimated due to the influence of soil crust covering the ground surface (Figure 3f; Figure 3f1 with d set as one). The effects of soil crust were reduced when d was set to 1.5 (Figure 3f2). However, cover was underestimated when d was set to two to eliminate the effects of soil crust (Figure 3f3). Flowers, especially white flowers in the mixed grassland, have large effects on extracting SDM (Figure 3f, Figure 8a,b). However, the effect of flowers cannot be eliminated by using higher d values (Figure 8b1: d = 1; Figure 8b2: d = 1.5; Figure 8b3: d = 1.7; Figure 8b4: d = 1.8; Figure 8b5: d = 1.9).

## 5. Conclusions

Our main conclusions are: (1) based on the linear relationship with NDVI, GV cover extracted from the method developed in this study (R^2^ = 0.846, *p* < 0.001) is superior to that from subjective visual estimation in the field (R^2^ = 0.711, *p* < 0.001), and the extracted GV cover is consistent with that estimated by Canopeo (i.e., a powerful tool for measuring GV cover in grassland). (2) The semi-automatic method of this study has high potential to extract SDM cover when the canopy cover (including both GV and SDM cover) is high or when the understory including the effects of litter, soil crust and bare soil, is limited. (3) Subjective visual estimation in the field tended to predict higher cover for both GV and SDM compared to that estimated from RGB images in this study.

## Figures and Tables

**Table 1:** Dominant species in upland, sloped, valley and disturbed communities.

Vegetation Community	Dominated Species
upland grassland	western wheatgrass (*Agropyron smithii Rydb.*)
	blue grama grass (*Bouteloua gracilis (HBK*) *Lang. ex Steud*.)
	needle-and-thread grass (*Stipa comata Trin. and Rupr.*)
valley grassland	northern wheatgrass (*Agropyron dasystachym*)
	western wheatgrass (*Agropyron smithii Rydb.*)
	with high density of shrub species
sloped grassland	northern wheatgrass (*Agropyron dasystachym*)
	western wheatgrass (*Agropyron smithii Rydb.*)
	needle-and-thread grass (*Stipa comata Trin. and Rupr.*)
	blue grama grass (*Bouteloua gracilis (HBK*) *Lang. ex Steud*.)
disturbed communities	crested wheatgrass (*Agropyron cristatum*)
	smooth brome (*Bromus inermis Layss.*)
	sweet clover (*Melilotus officinalis*)

**Table 2:** Descriptive statistics for the coverage data based on visual estimation.

ID	Vegetation Community	Green Cover (GV)		Standing Dead Matter (SDM) Cover		Non-Photosynthetic Vegetation (NPV) Cover	
		Mean	Standard Deviation (STD)	Mean	STD	Mean	STD
1	upland grassland	48.85	13.02	7.75	8.50	39.60	8.77
2	upland grassland	42.15	14.72	31.85	9.18	51.85	11.89
3	upland grassland	48.24	6.91	7.14	5.82	45.90	6.36
4	upland grassland	40.55	9.61	16.50	9.75	39.40	19.37
5	valley grassland	42.85	14.04	24.75	11.97	27.30	12.21
6	valley grassland	37.95	12.39	24.75	11.53	34.10	14.97
7	valley grassland	45.71	16.30	26.52	18.90	40.71	20.08
8	sloped grassland	28.25	13.35	15.35	16.33	30.30	22.66
9	sloped grassland	35.35	10.92	26.45	14.33	44.65	20.05
10	sloped grassland	39.71	10.62	23.57	14.24	46.71	12.36
11	sloped grassland	44.95	7.35	15.95	8.75	39.90	11.71
12	sloped grassland	38.95	8.96	9.67	5.18	32.19	11.60
13	disturbed communities	62.40	18.86	12.50	7.86	12.50	7.86
14	disturbed communities	49.76	11.34	14.76	10.30	14.76	10.30

**Table 3:** Data exploration for constant $g_{1},\;g_{2}$ for different species and conditions.

ID	Vegetation Community	Species	Condition	*g*_1_				*g*_2_			
				MIN	MAX	MEAN	STD	MIN	MAX	MEAN	STD
1	disturbed community	smooth brome/forb	normal condition	60.18	276.81	139.94	41.26	60.53	533.56	199.87	60.55
2	disturbed community	smooth brome	normal condition	60.23	268.79	133.36	38.97	60.24	517.52	192.35	65.15
3	disturbed community	smooth brome/forb	normal condition	60.18	260.76	124.73	36.37	60.33	577.69	226.32	67.68
4	disturbed community	smooth brome	high exposure	38.12	224.66	94.36	23.41	60.15	545.60	267.27	55.50
5	disturbed community	sweet clover	normal condition	64.22	649.91	198.42	74.89	66.13	1014.98	434.55	167.75
6	disturbed community	sweet clover	normal condition	64.11	328.96	170.97	51.25	66.03	776.71	333.94	98.38
7	sloped grassland	needle and thread/northern wheat grass	high exposure	32.09	577.69	119.27	50.33	60.47	774.27	201.43	83.10
8	sloped grassland	western wheat grass/needle and thread	high exposure	36.11	284.84	106.99	33.44	60.36	585.72	147.92	59.82
9	sloped grassland	needle and thread	senesced grass	40.12	196.58	79.25	18.26	60.33	469.38	180.69	63.62
10	sloped grassland	needle and thread/western wheat grass	high exposure	44.13	517.52	117.68	50.47	60.23	786.31	248.96	85.93
11	sloped grassland	June grass/needle and thread/forb	high exposure, senesced grass	44.13	244.72	96.79	27.01	60.34	625.84	256.16	81.21
12	sloped grassland	June grass/western wheat grass	normal condition	61.02	284.84	117.41	38.13	60.15	501.47	155.81	60.03
13	sloped grassland	June grass/western wheat grass	normal condition	60.39	280.82	129.48	41.20	61.11	509.49	165.86	63.25
14	sloped grassland	June grass/western wheat grass/forb	normal condition	61.54	252.74	106.01	33.76	60.02	469.38	132.68	54.99
15	upland grassland	northern wheat grass	high exposure, senesced grass	36.11	445.31	127.37	50.69	60.03	710.47	299.97	83.84
16	upland grassland	June grass/northern wheat grass	senesced grass	40.12	296.87	108.04	34.27	62.11	585.72	185.94	77.32
17	upland grassland	northern wheat grass/needle and thread	high exposure, senesced grass	36.11	240.71	100.90	28.54	63.12	557.64	264.67	60.56
18	upland grassland	western wheat grass/needle and thread	normal condition	61.27	629.85	132.76	55.72	60.01	826.42	281.67	96.35
19	valley grassland	western wheat grass	bluish leaves	61.22	224.66	86.35	22.01	32.09	453.33	120.11	43.96
20	valley grassland	crested wheat grass	bluish leaves	60.14	260.76	116.32	34.09	36.11	561.65	199.28	74.63
21	valley grassland	western wheat grass/sagebrush	bluish leaves	60.18	256.75	95.04	25.59	40.12	533.56	157.27	65.15
22	valley grassland	smooth brome/forb	normal condition	66.13	300.88	162.84	47.67	64.19	429.26	177.65	55.57
23	valley grassland	northern wheat grass	normal condition	61.22	276.81	114.85	36.49	60.23	485.42	137.31	55.02
24	valley grassland	western wheat grass/Little blue steam	normal condition	62.12	216.64	90.98	23.61	60.01	477.40	168.68	51.78
25	valley grassland	western wheat grass/forb	bluish leaves	60.28	232.68	90.59	22.40	40.12	272.80	125.49	41.29

## Supplementary Materials

The following are available online at https://www.mdpi.com/1424-8220/20/23/6870/s1, Supplementary S1: explanation of the parameters in the python script for extracting green cover and standing dead cover from field--taken RGB images; Supplementary S2: RGB pictures for each quadrat in 14 sites; Supplementary S3: Sample RGB pictures for data exploration of constants $g_{1},\;g_{2}$ in Table 3; Python script, ArcToolbox.
