# Supplementary material for: A Semi-Automated Method to Extract Green and Non-Photosynthetic Vegetation Cover from RGB Images in Mixed Grasslands
Source: Sensors (Basel). 2020 Dec 1;20(23):6870. doi: 10.3390/s20236870 (PMC7731437; doi:10.3390/s20236870)
Supplement: Supplementary file 1 [file sensors-20-06870-s001.zip › supplementary S3.pdf]

Supplementary S3

id	Vegetation community	Sample RGB picture
1	disturbed community	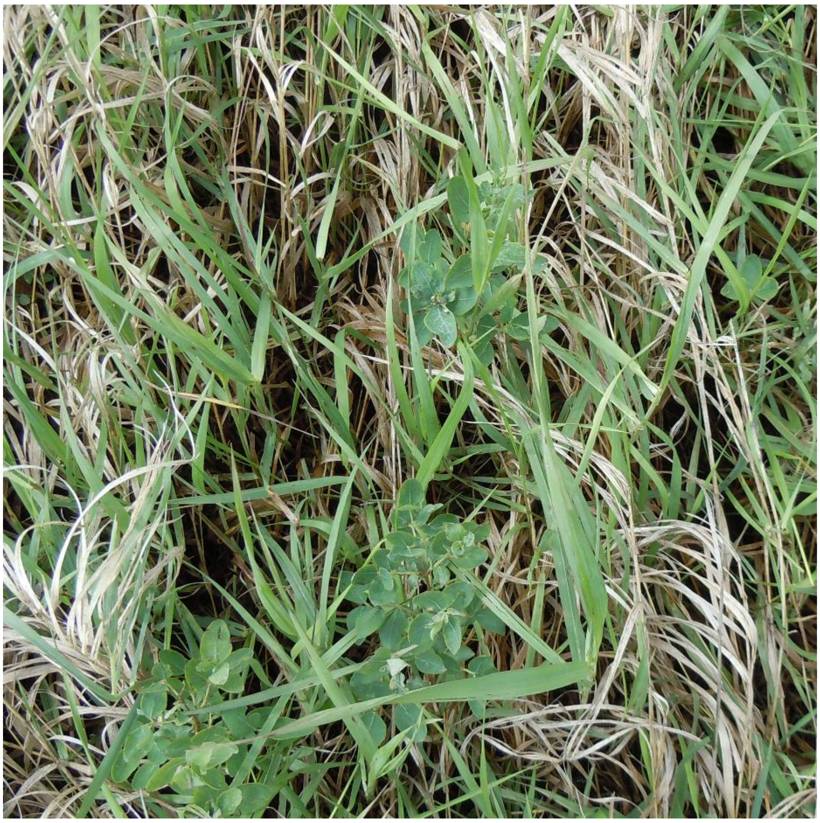
2	disturbed community	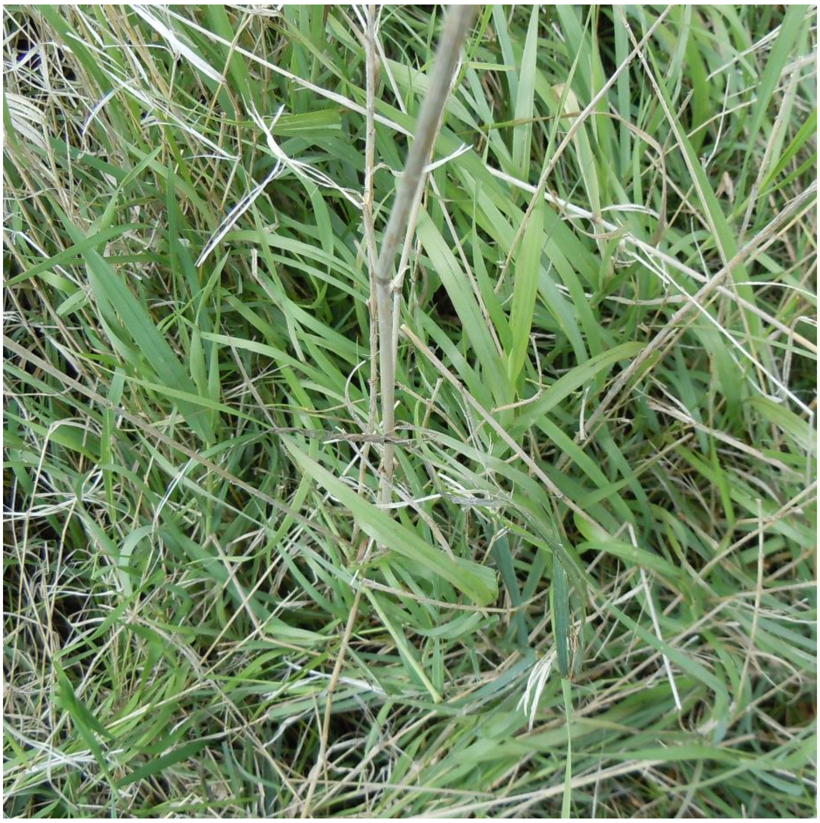

3	disturbed community	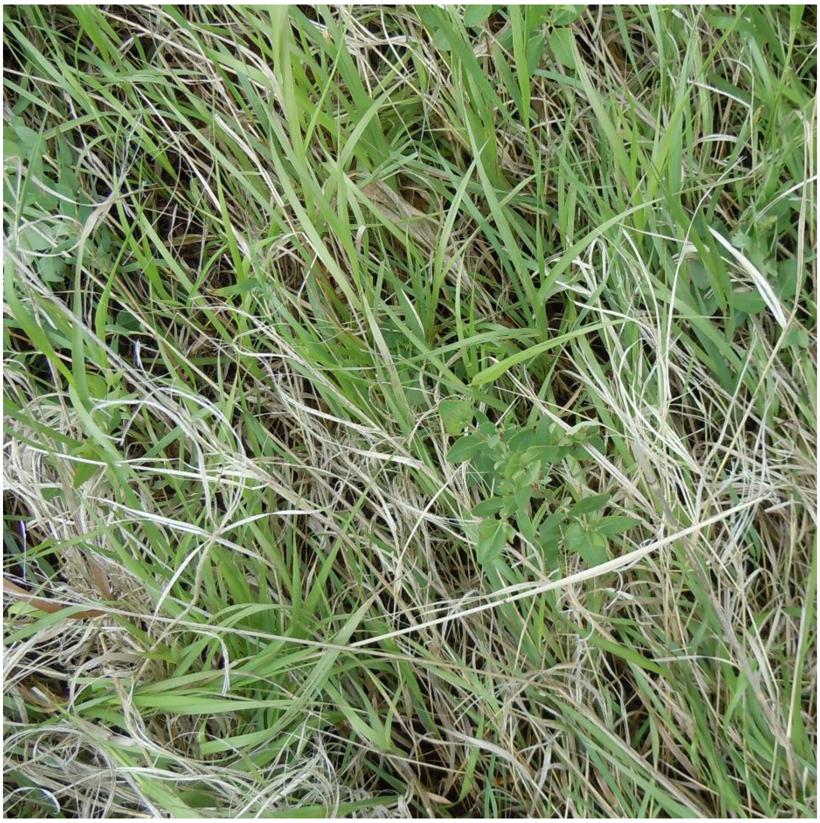
4	disturbed community	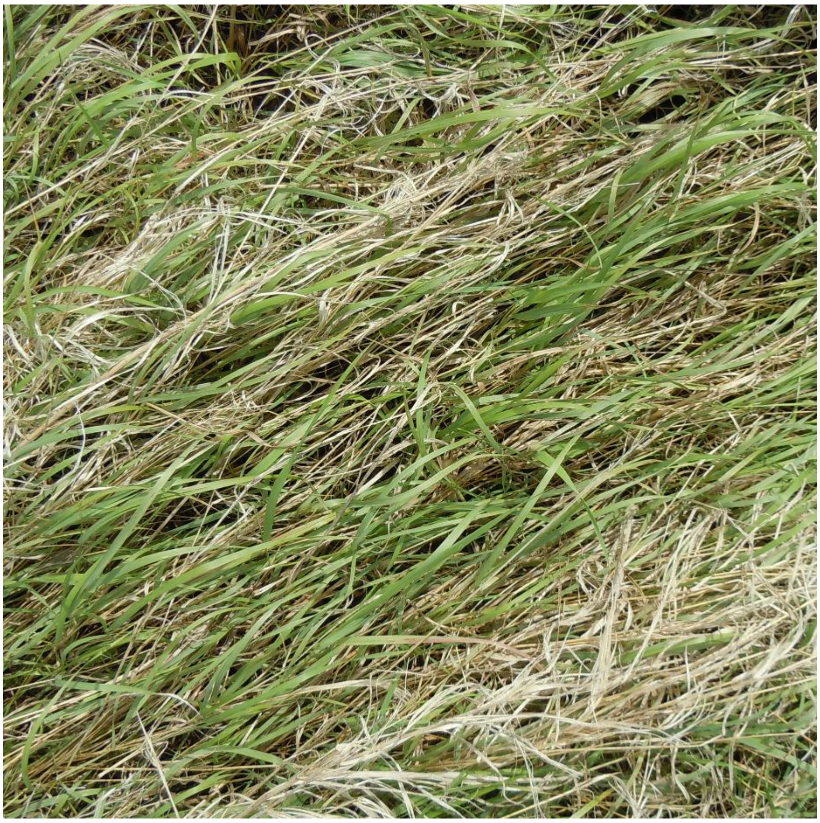

5	disturbed community	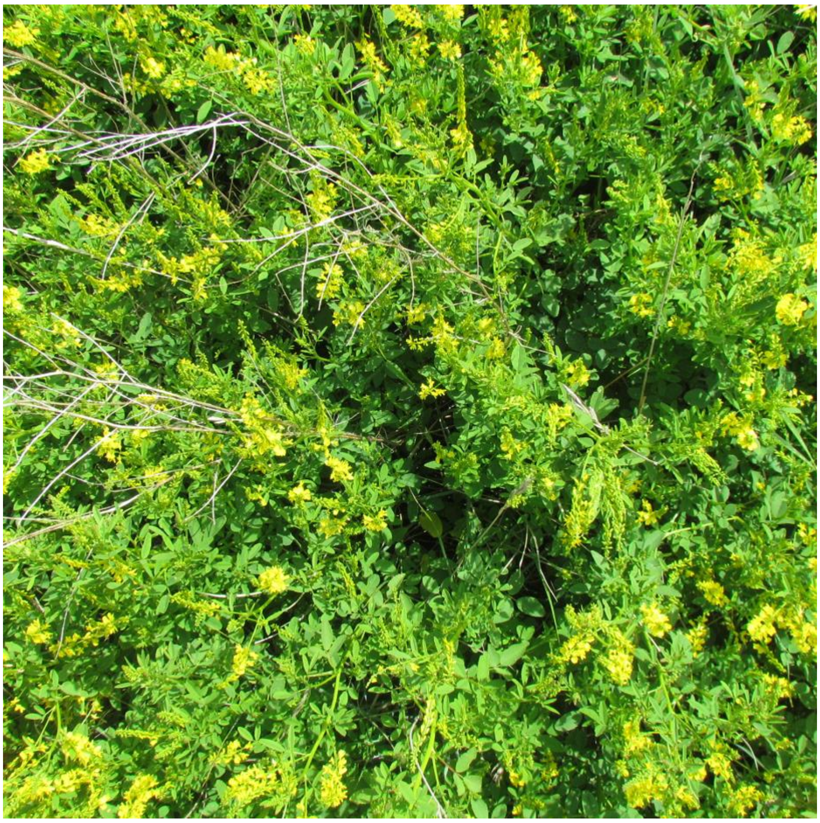
6	disturbed community	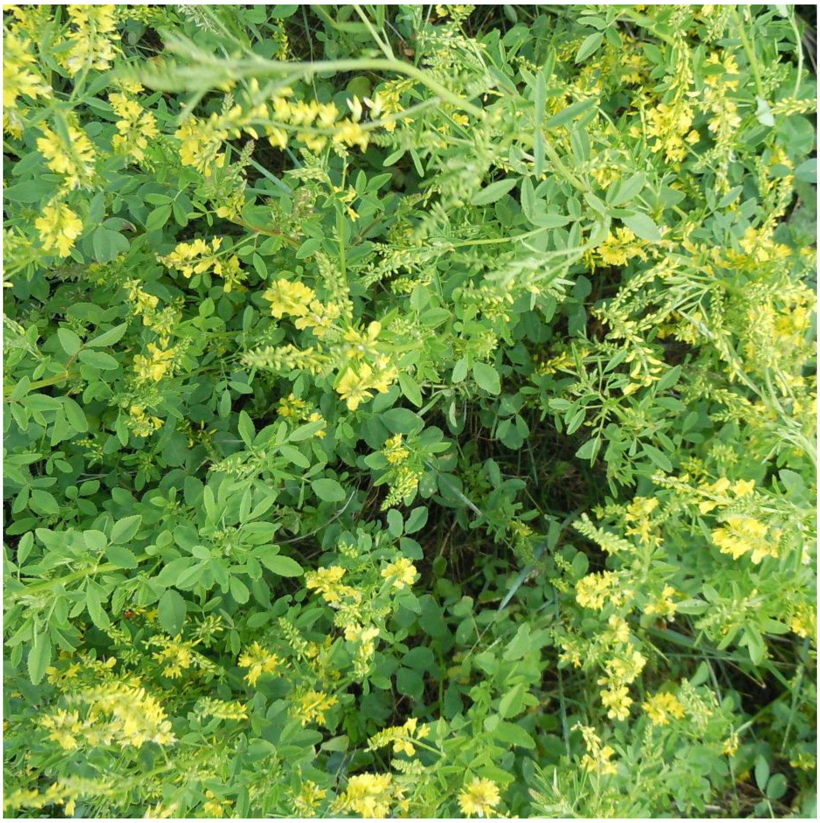

7	sloped grassland	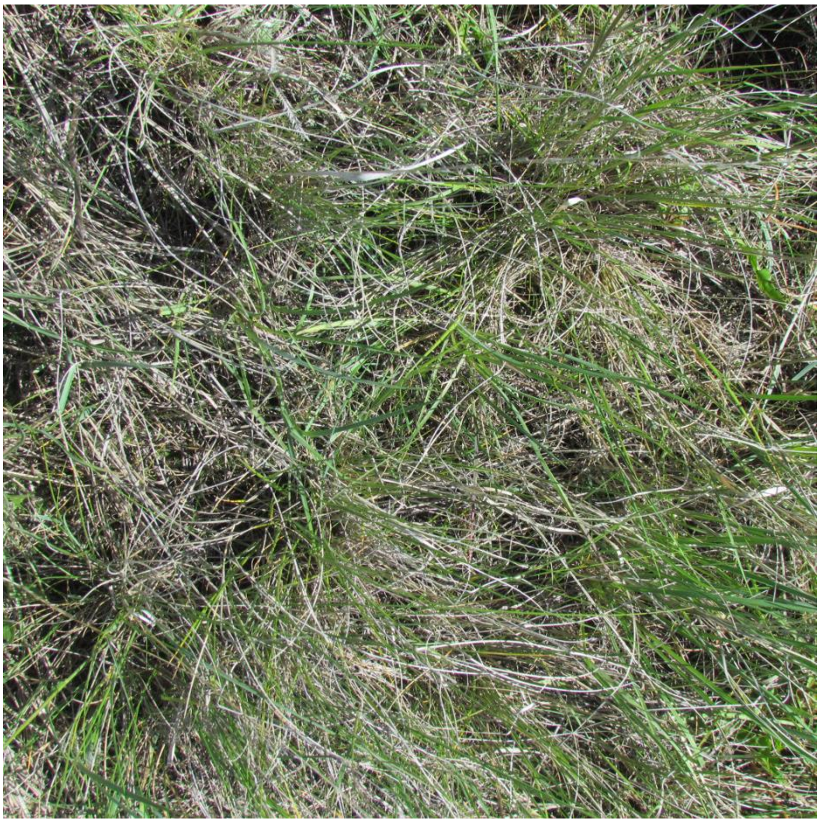
8	sloped grassland	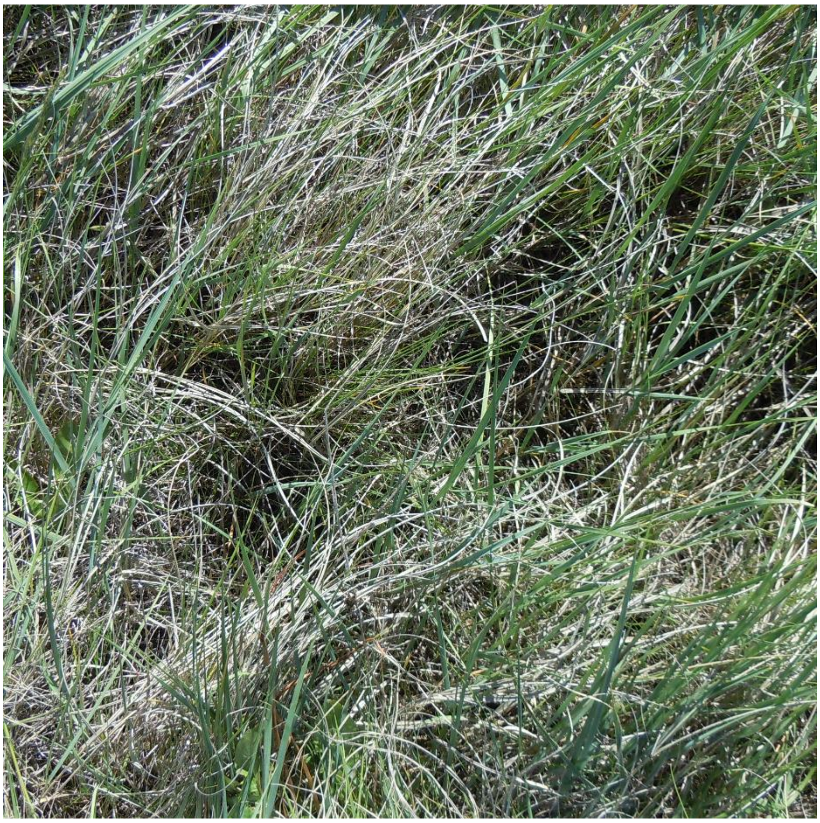

9	sloped grassland	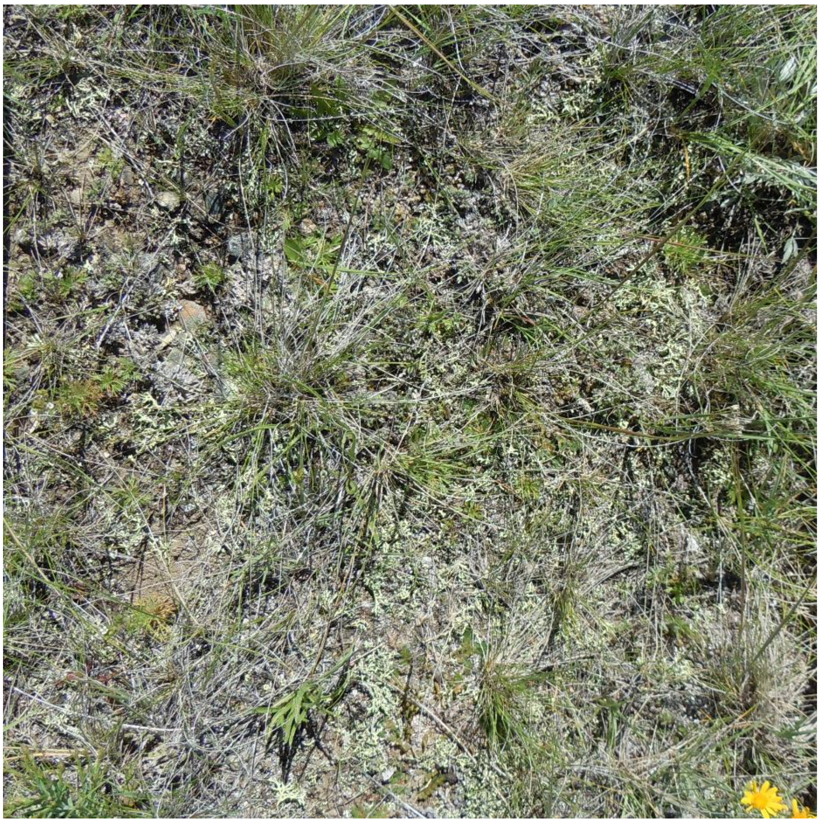
10	sloped grassland	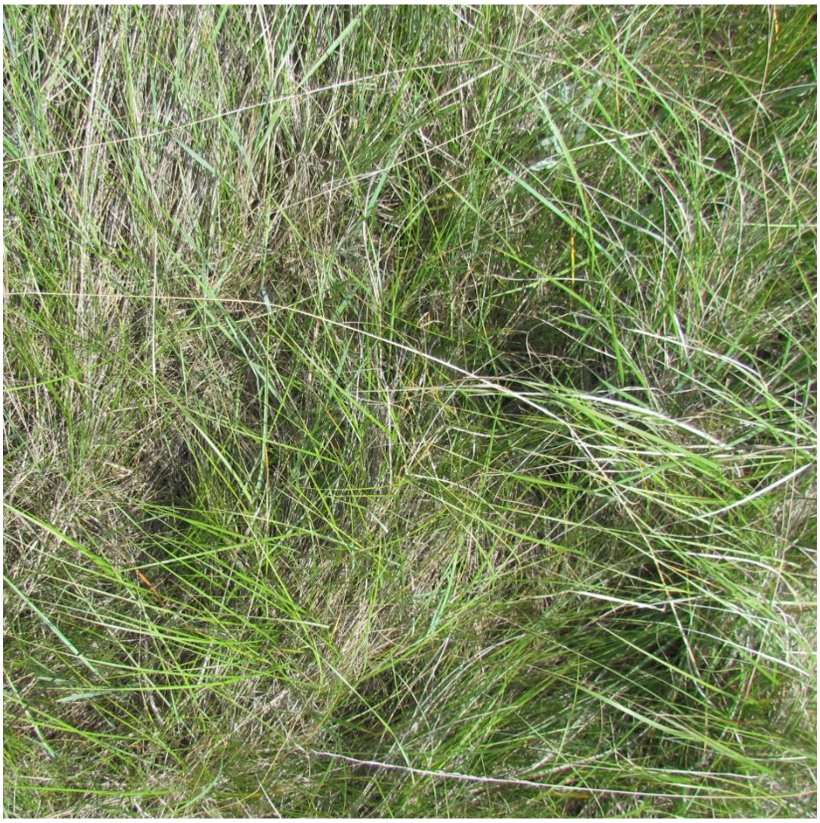

11	sloped grassland	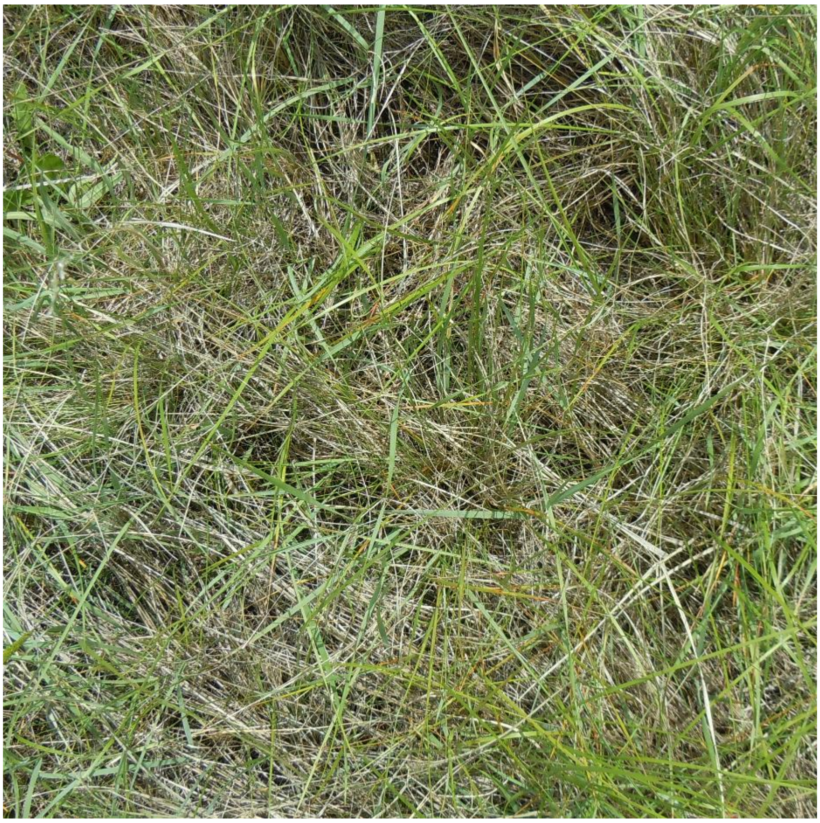
12	sloped grassland	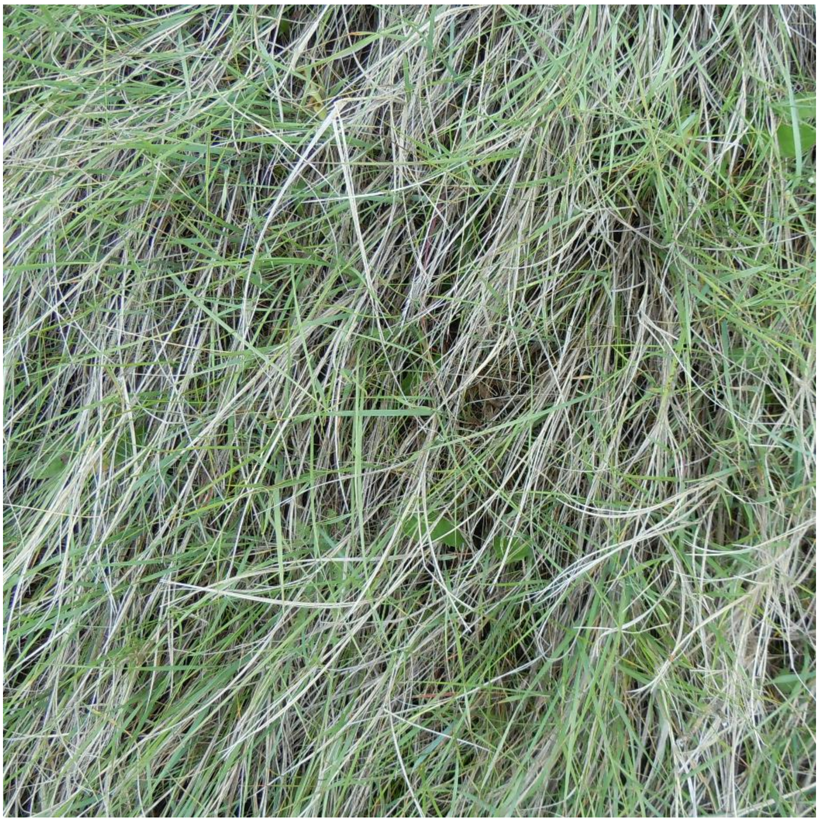

13	sloped grassland	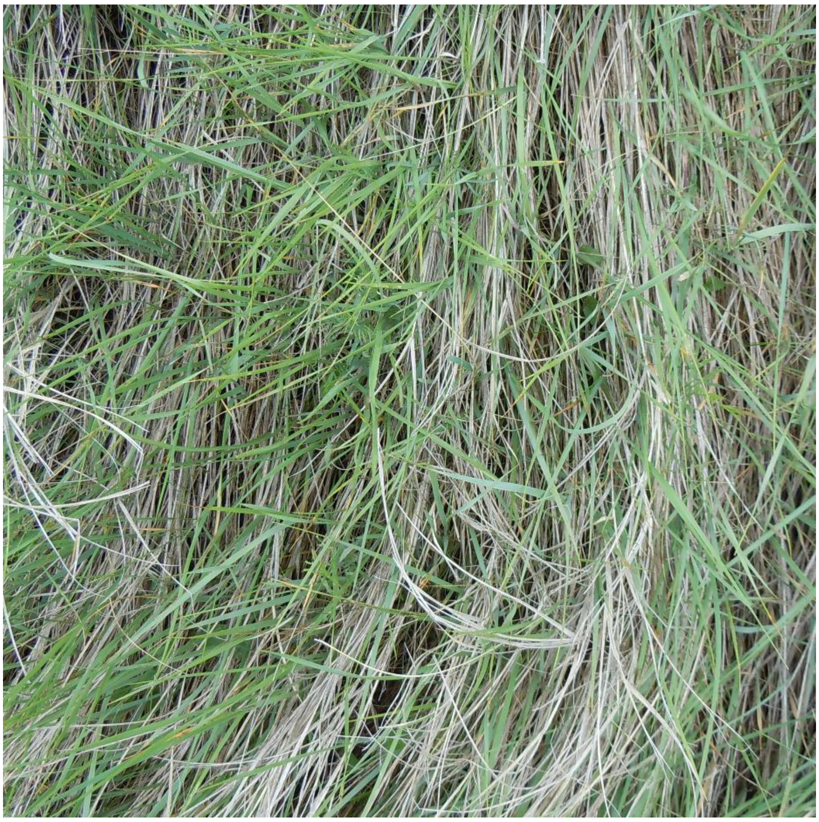
14	sloped grassland	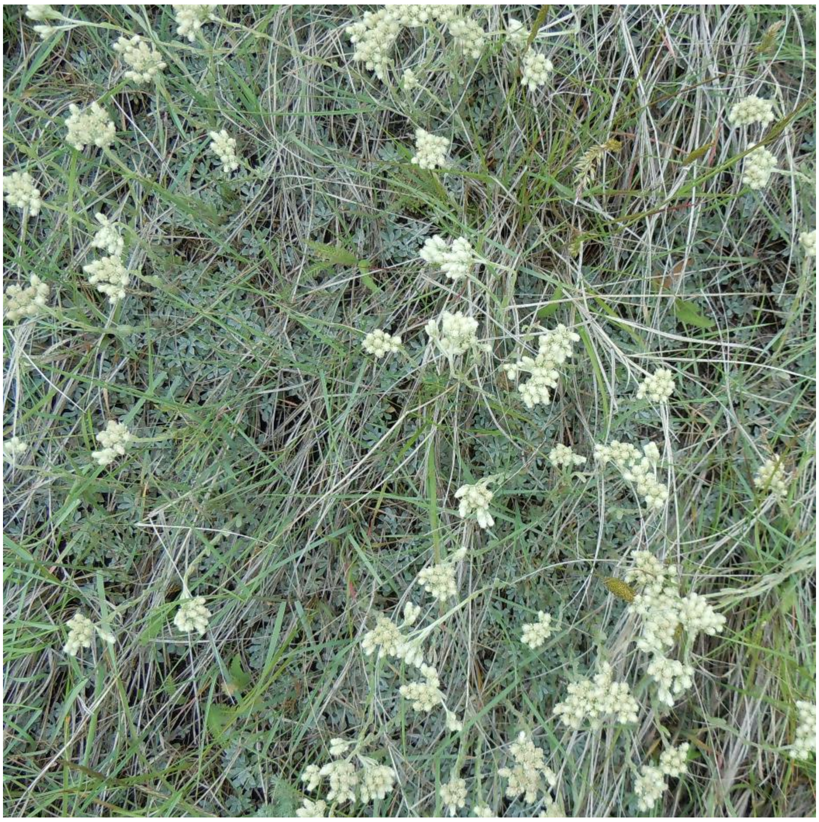

15	upland grassland	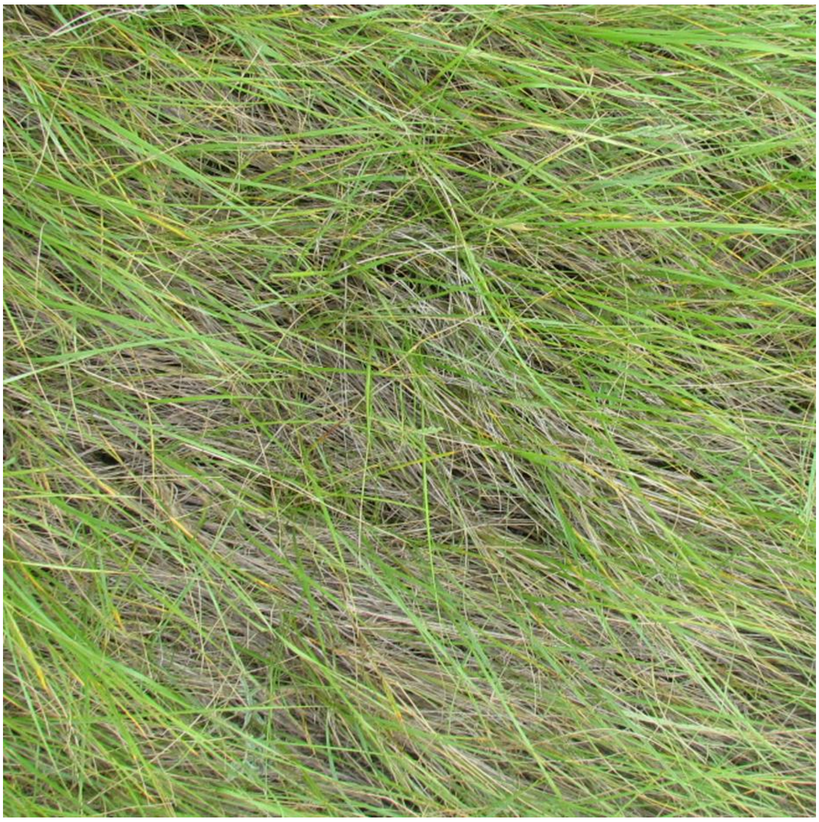
16	upland grassland	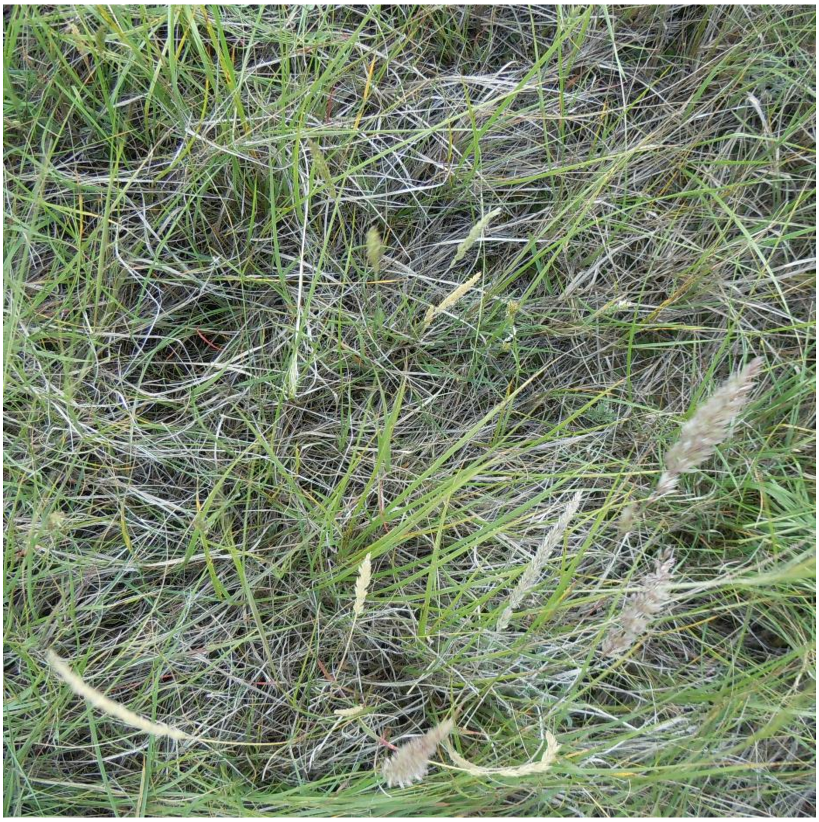

17	upland grassland	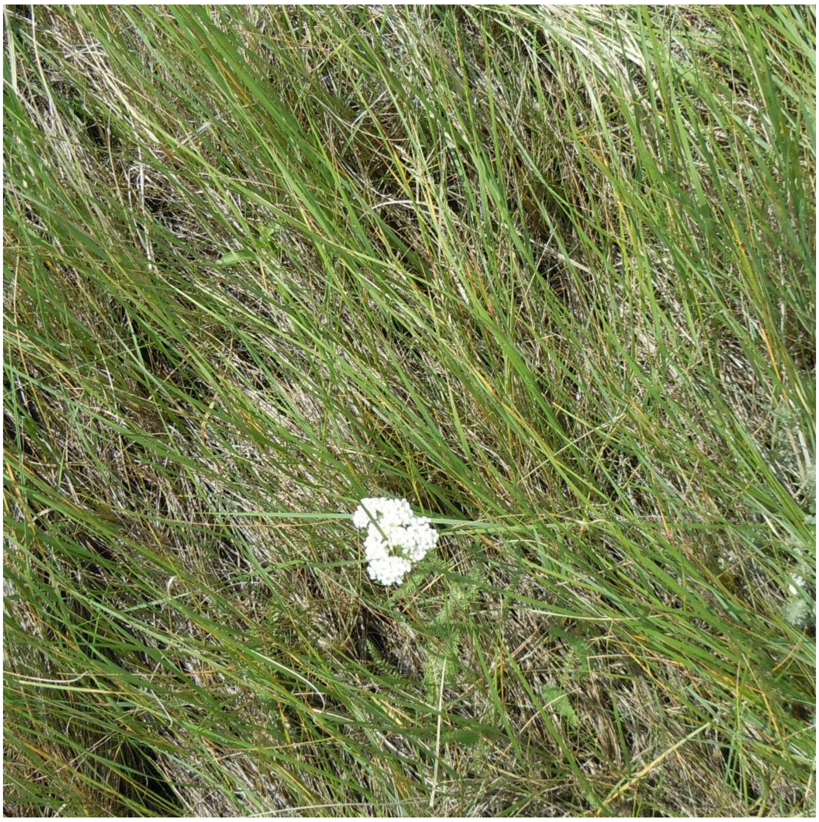
18	upland grassland	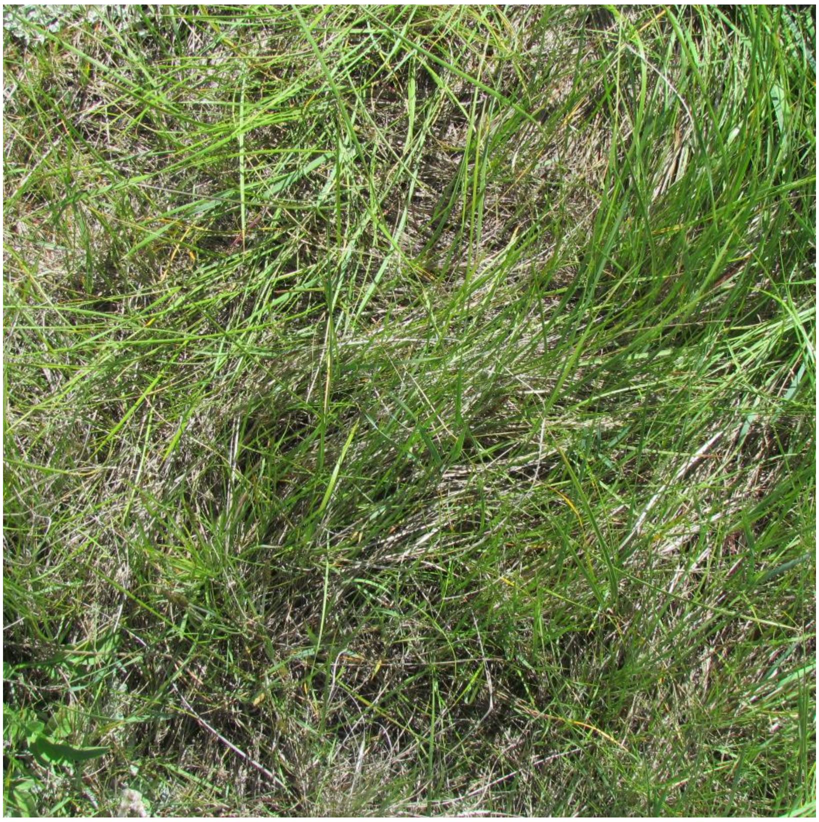

19	valley grassland	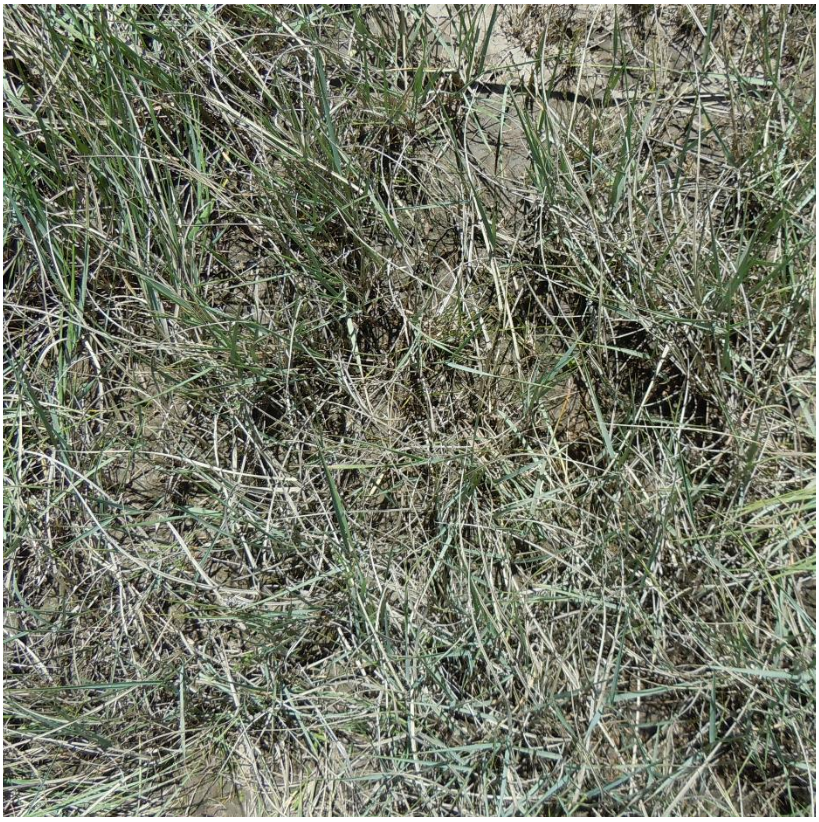
20	valley grassland	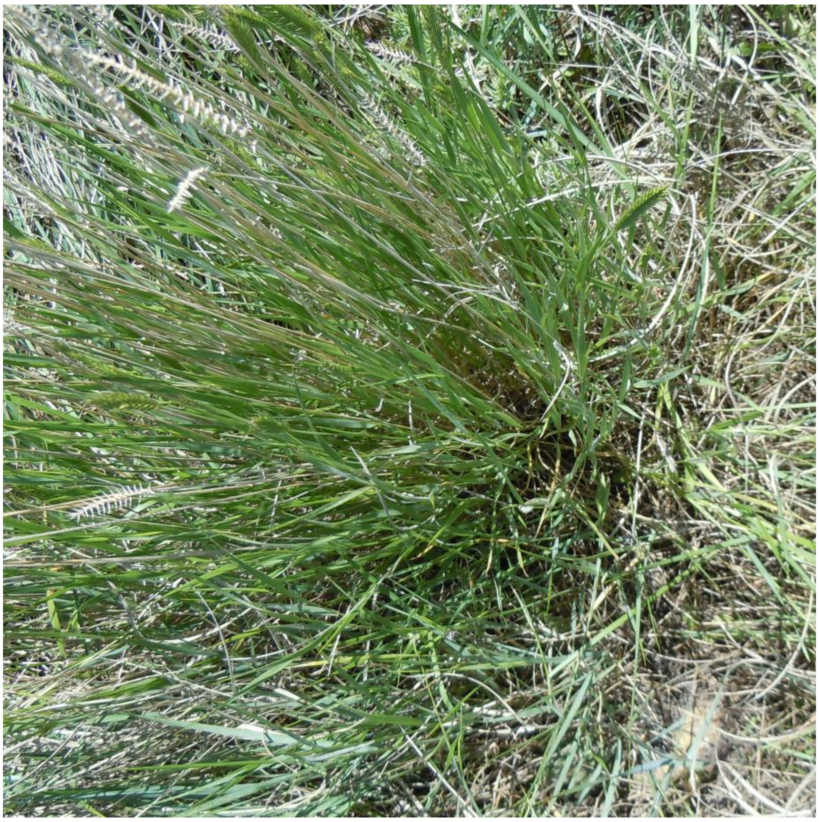

21	valley grassland	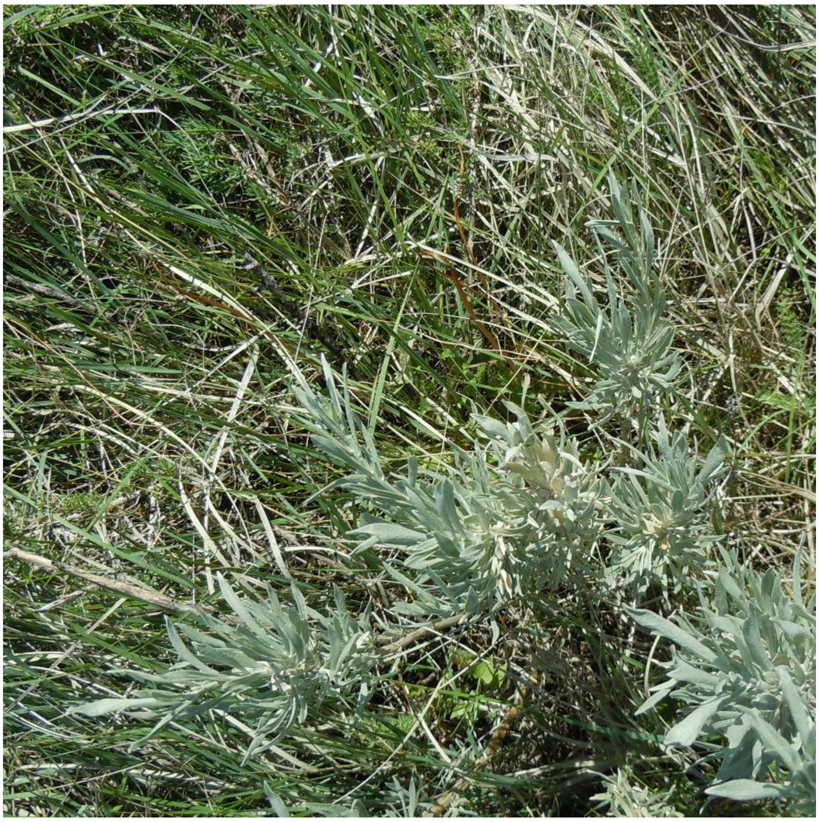
22	valley grassland	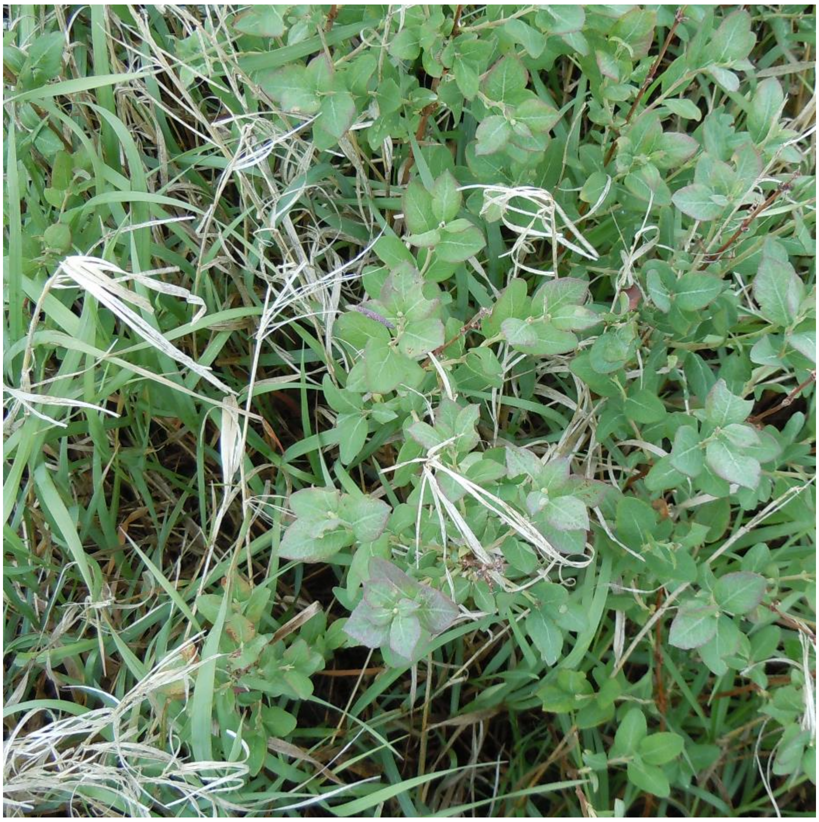

23	valley grassland	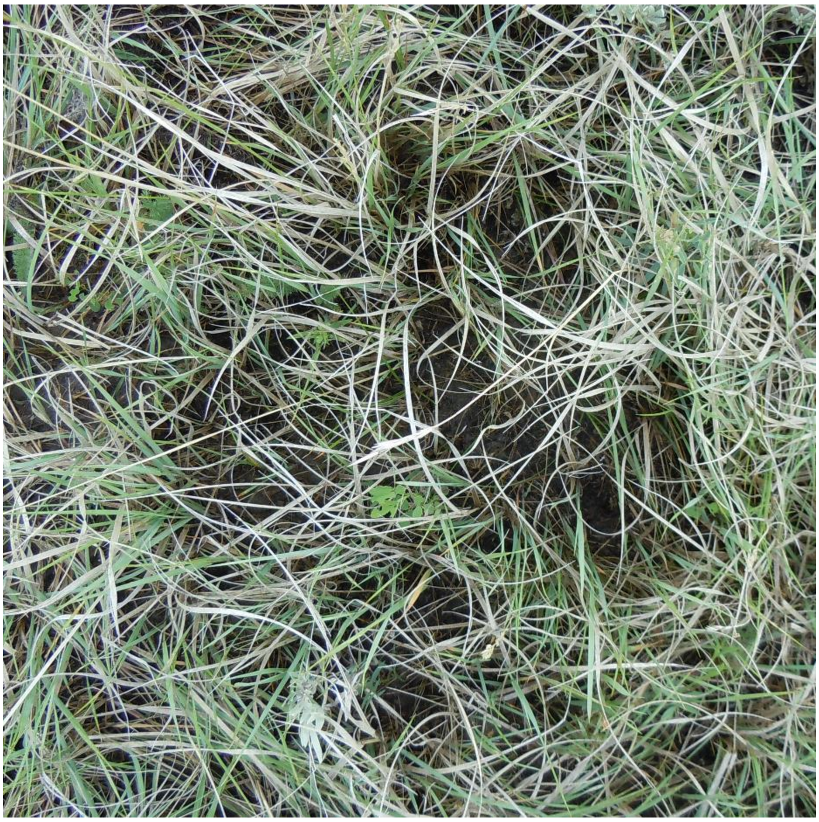
24	valley grassland	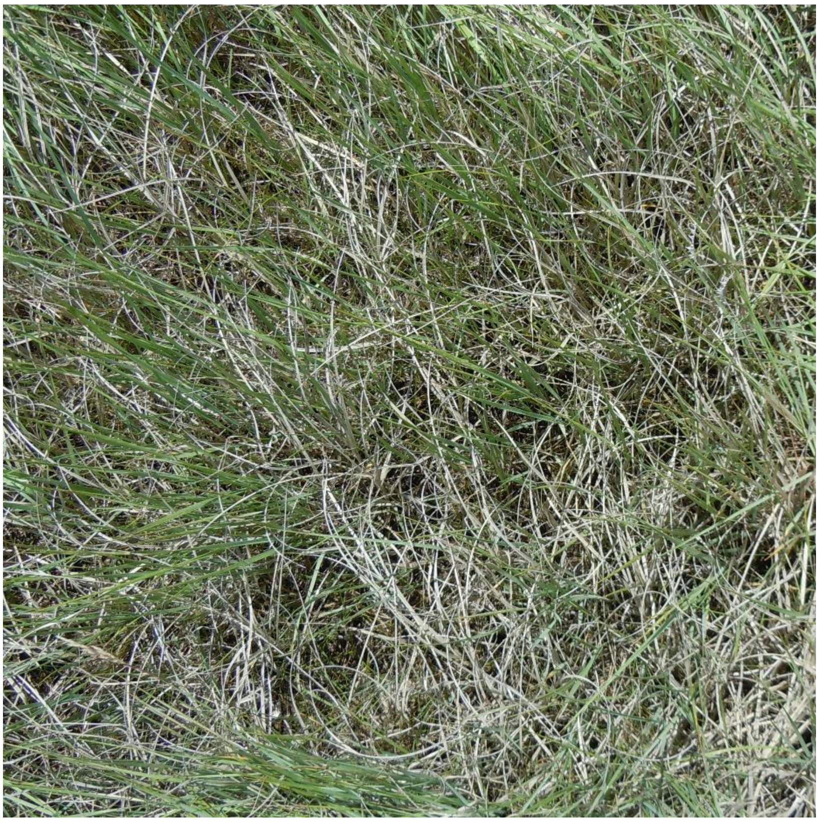

25	valley grassland	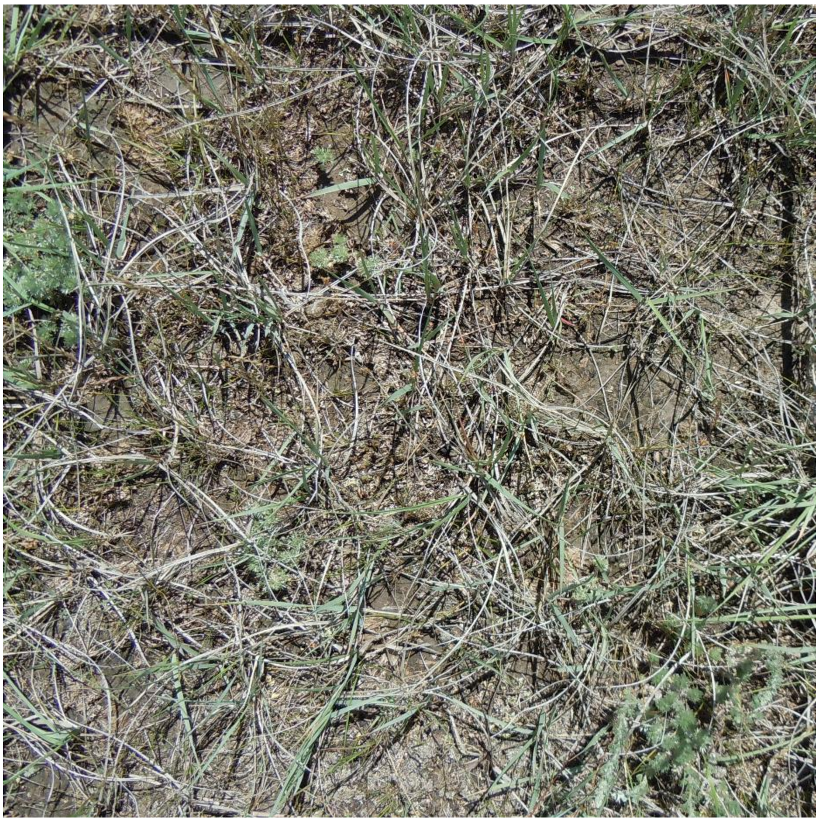
