# Supplementary material for: A Semi-Automated Method to Extract Green and Non-Photosynthetic Vegetation Cover from RGB Images in Mixed Grasslands
Source: Sensors (Basel). 2020 Dec 1;20(23):6870. doi: 10.3390/s20236870 (PMC7731437; doi:10.3390/s20236870)
Supplement: Supplementary file 1 [file sensors-20-06870-s001.zip › supplementary S1.docx]

**Supplementary S1**

**The interface of the tool when import the python script developed in this study to ArcGIS**


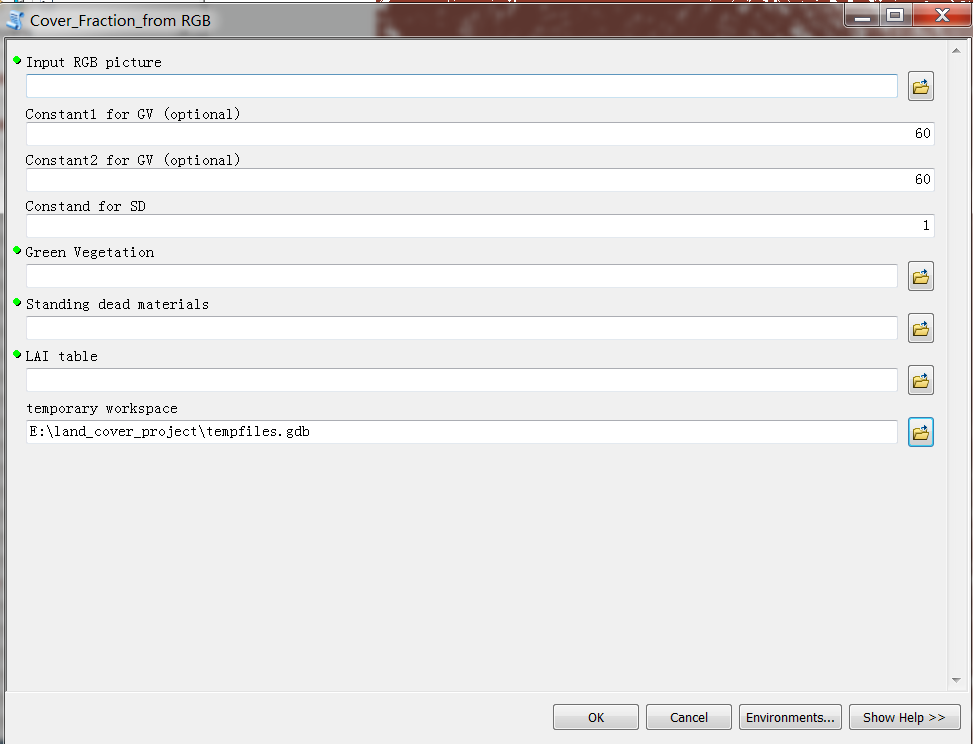


**1. Inputs of the tool**

**1.1. Input RGB picture**

The input RGB picture is the cropped picture including three visible bands taken in the field with a commercial camera.

**1.2. Constant1 for GV (optional) & Constant2 for GV (optional)**

In most of the situations, both Constant1 and Constant2 for GV (green cover extraction) are set as the default value 60 (Figure 1). The value for Constant1 and Constant2 should be altered depending on different vegetation types, phenology stage, soil crust and the light conditions when taking the RGB pictures.


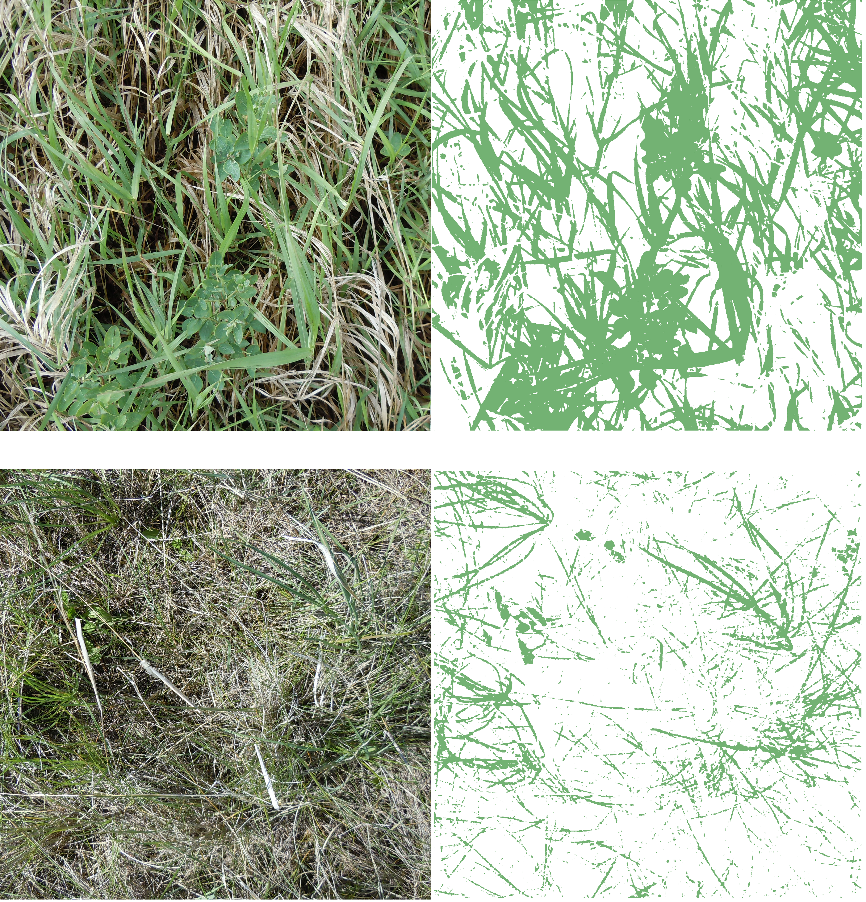


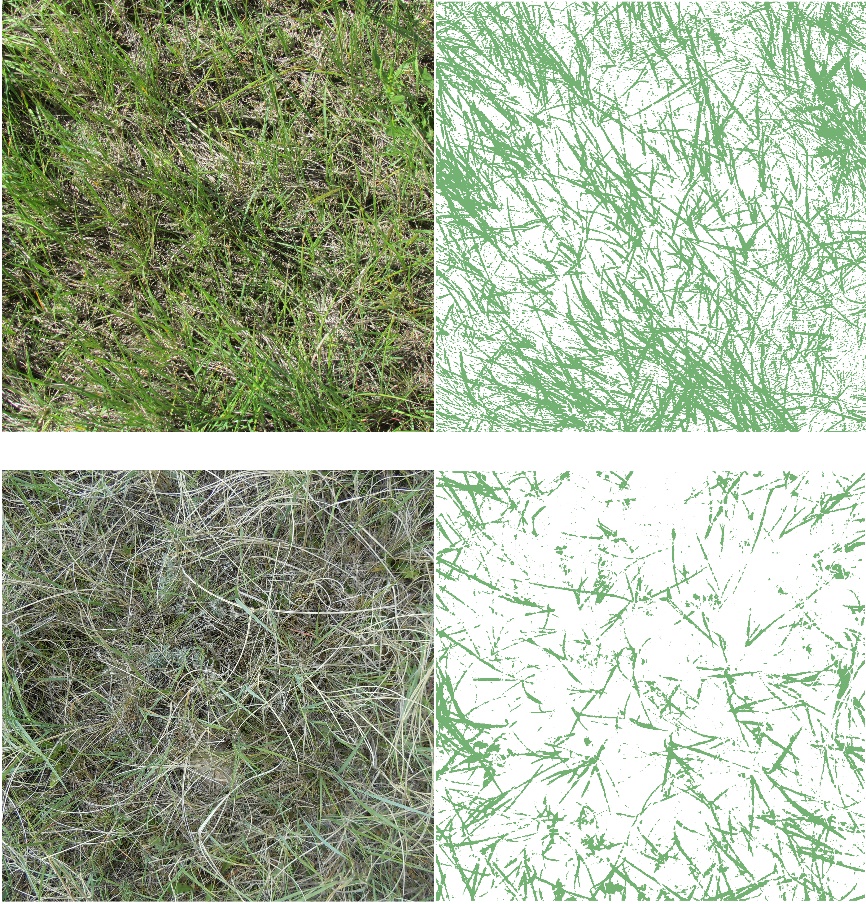


**Figure 1.** Extracted green vegetation from RGB pictures

When the vegetation is already in the senescence stage (Figure 2), constant1 need to be set as a value lower than the default value 60 (e.g. 30 or 40).


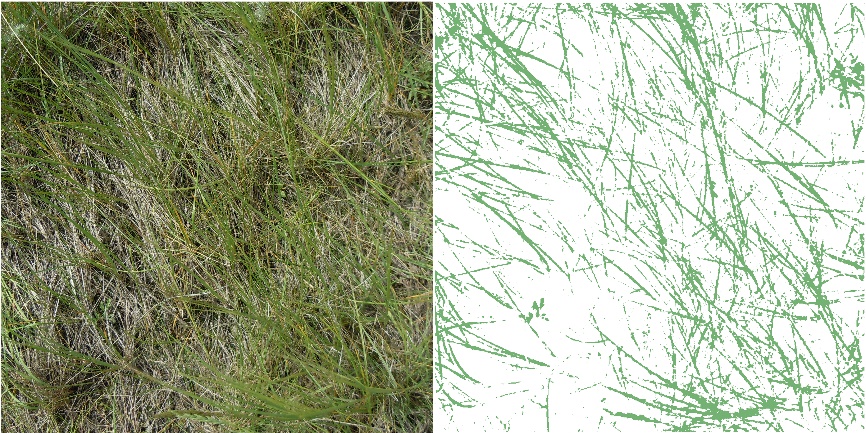


**Figure 2.** Vegetation in the senescence stage (constant1 is 30)

When the RGB picture contains green moss (soil crust with green color), Constant1 should be set higher than Constant2 because the difference between green band and blue band is far larger than the different between green band and red band for green moss (Figure 3).


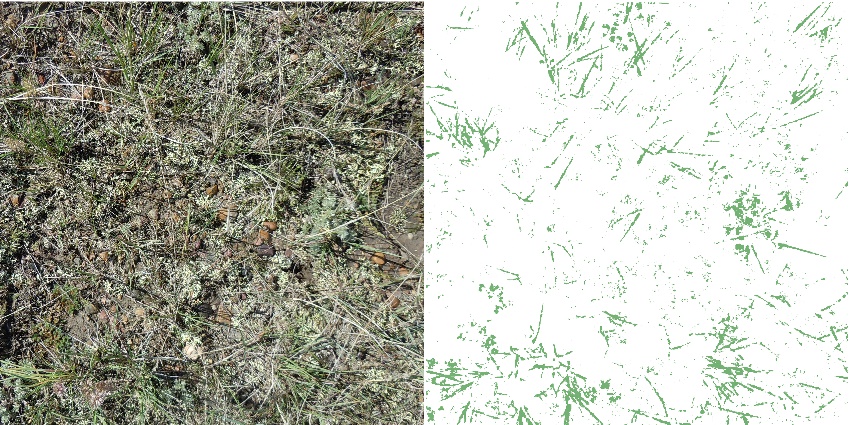


**Figure 3.** RGB picture with green moss (Constant1 and Constant2 are 60 and 40 respectively)

Sage and western wheatgrass show a little bluish color in RGB pictures. In this case, Constant2 should be set as a negative value and Constant1 should be a little lower than default value (Figure 4).

In addition, both constant need to be set lower than the default value 60 when there is too much exposure while taking the RGB pictures in the field.


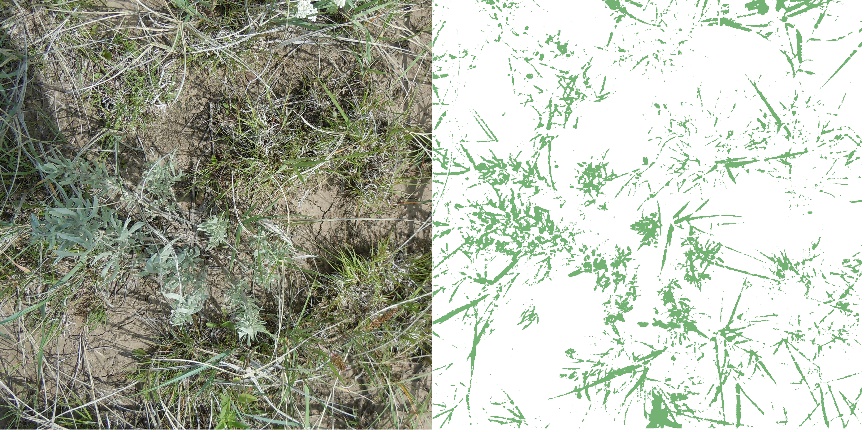


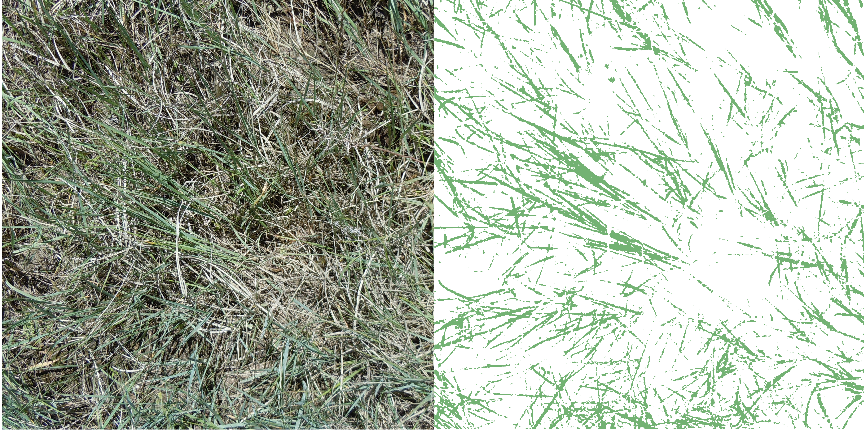


**Figure 4.** Extracting sage and western wheatgrass(Constant1 and Constant2 are 40 and 32 respectively)

**1.3. Constant for SD**

The Constant for SD is designed to separate standing dead materials from litter and light soil background and the default value of this constant is 1 (Figure 5).

Litter has big effects on the accuracy for extracting standing dead materials because it has similar spectral signal with standing dead materials. However, litter is a bit darker than standing dead material of all the three bands because light exposure is different for the canopy and understory, and the color tong of litter becomes darker when it starts to get decomposition. To reduce the impact of litter when extracting standing dead materials, the Constant for SD should be set higher than default value (Figure 6).

Dry bare soil with light color tong is another issue for extracting standing dead materials when the canopy color is low. The errors caused by light soil background are possible to be reduced by setting the higher value of the Constant for SD (Figure 7).


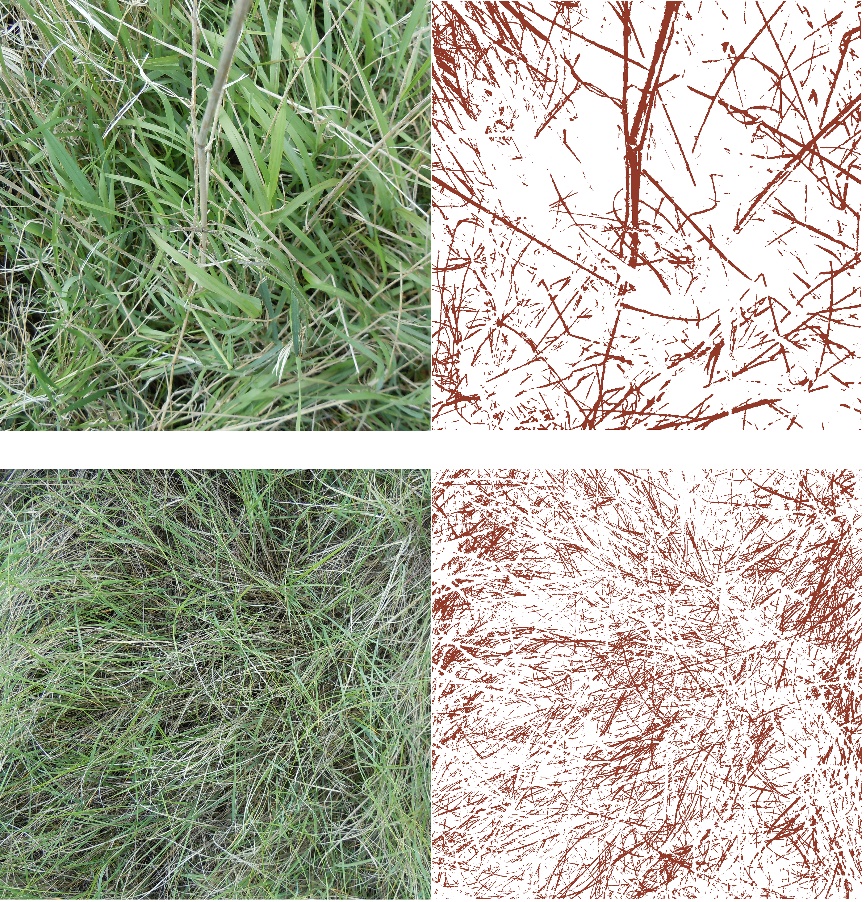


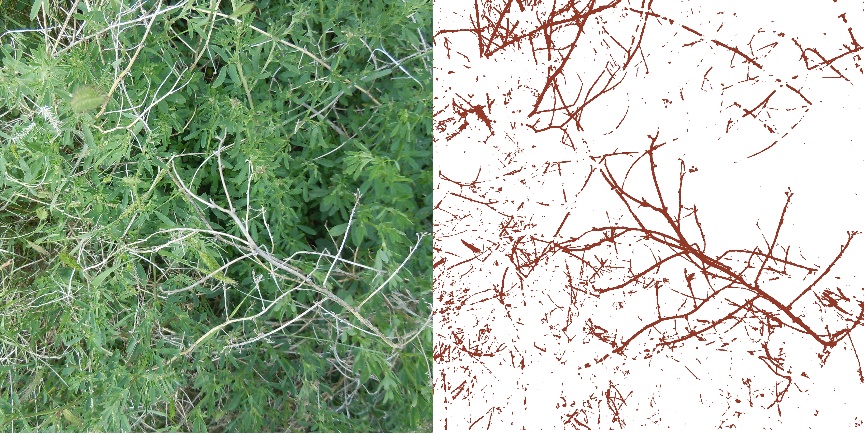


**Figure 5.** Extracted standing dead materials from RGB pictures


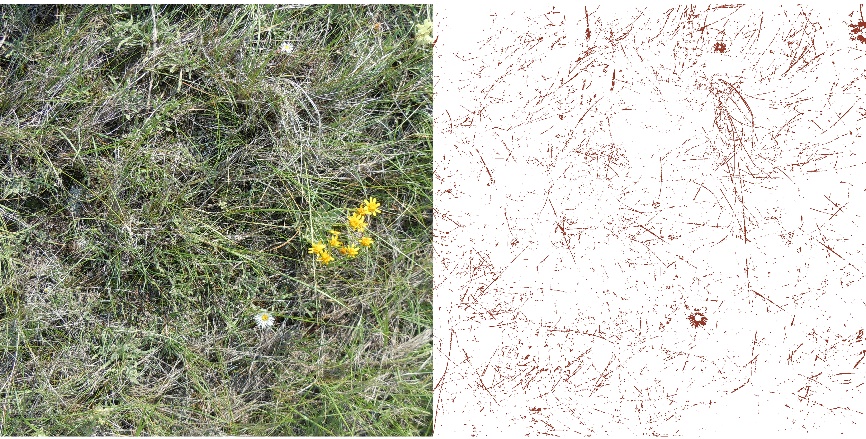


**Figure 6.** Litter effects while extracting standing dead materials (Constant for SD is set as 1.7)


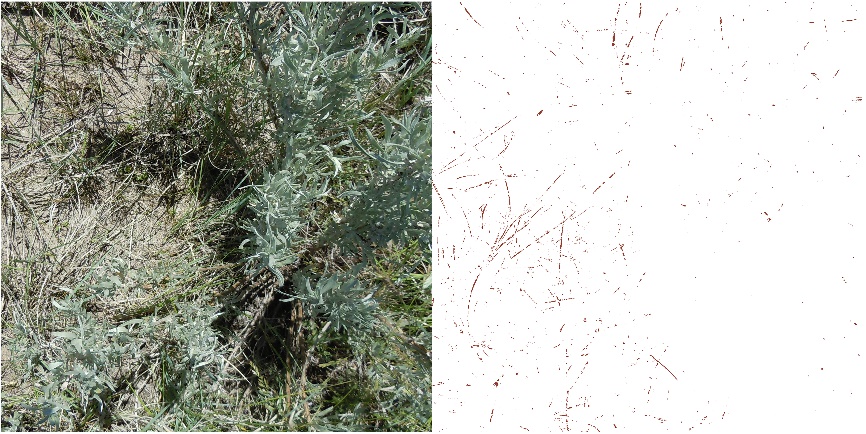


**Figure 8.** Light soil effects while extracting standing dead materials (Constant for SD is set as 2)

The standing dead materials in the lower canopy have darker color tong than those in the upper canopy, thus, the standing dead in the lower canopy might be treated as litter to be excluded in the output of standing dead cover (Figure 9). In this specific case, the Constant for SD should be lower than the default value 1 (Figure 9).


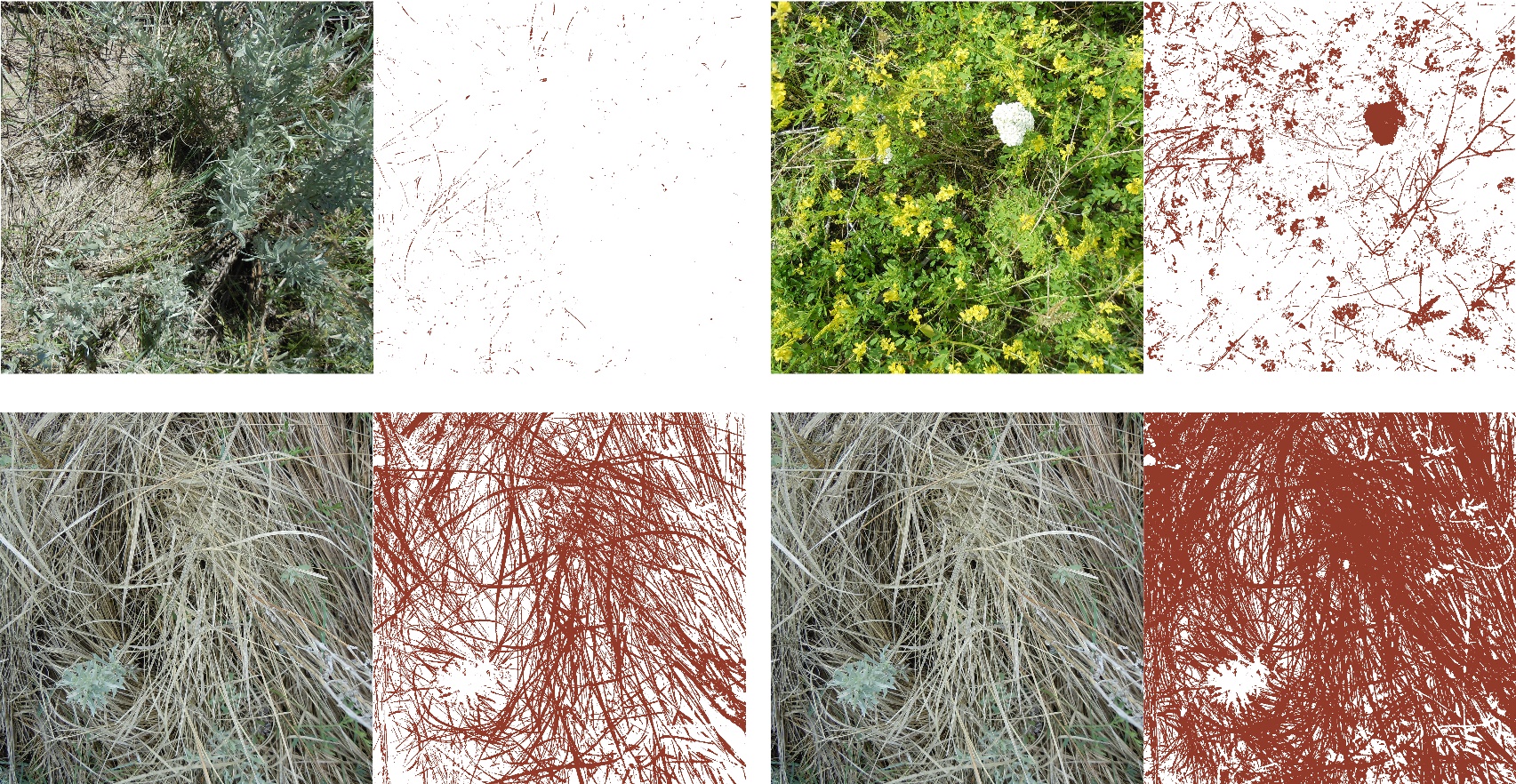

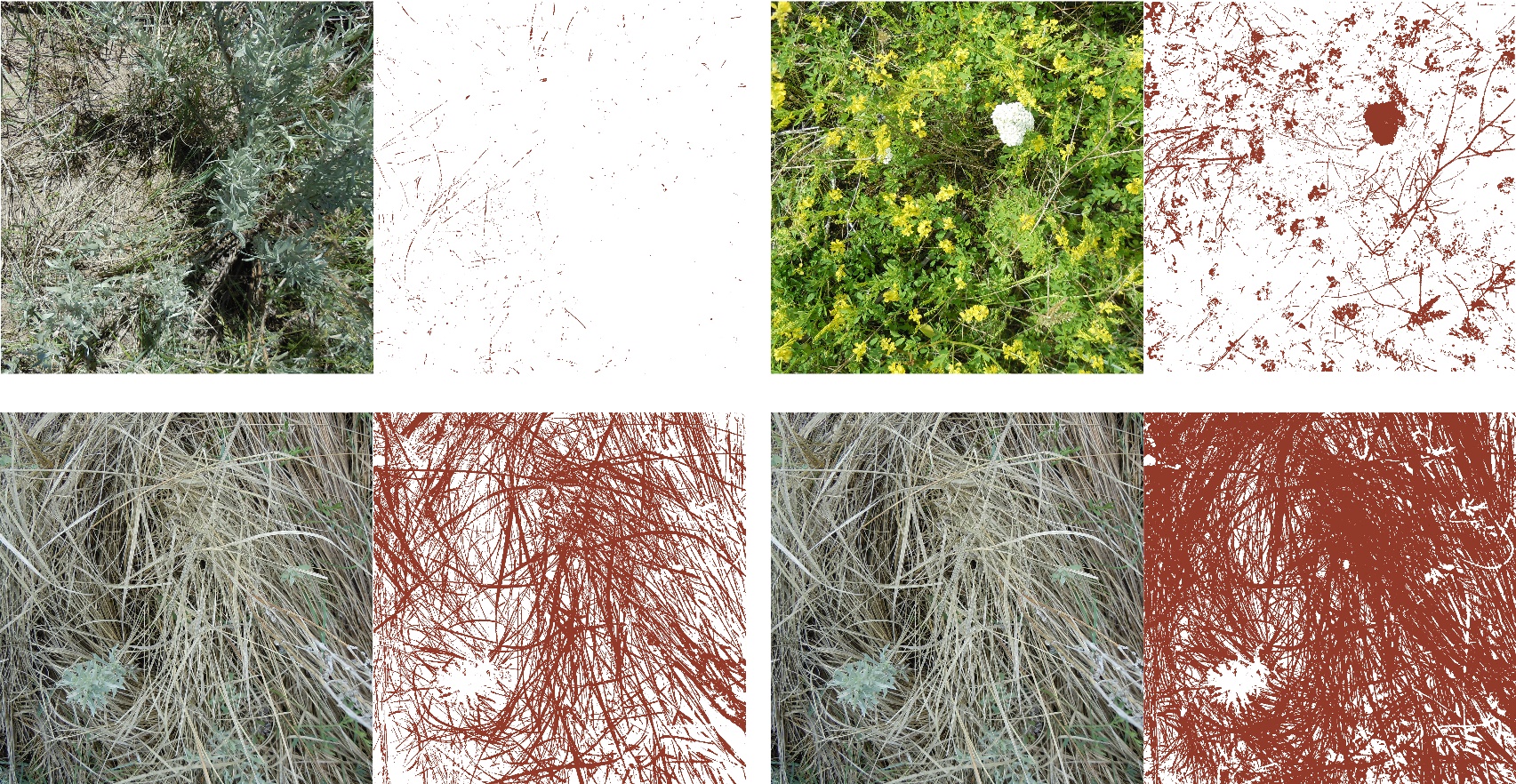


**Figure 9.** Standing dead material with complex canopy structure (Constant for SD is 1 for the middle picture and 0.5 for the right picture)

In addition, white flowers instead of yellow flowers in the mixed grassland have great effects on standing dead extraction (Figure 10).


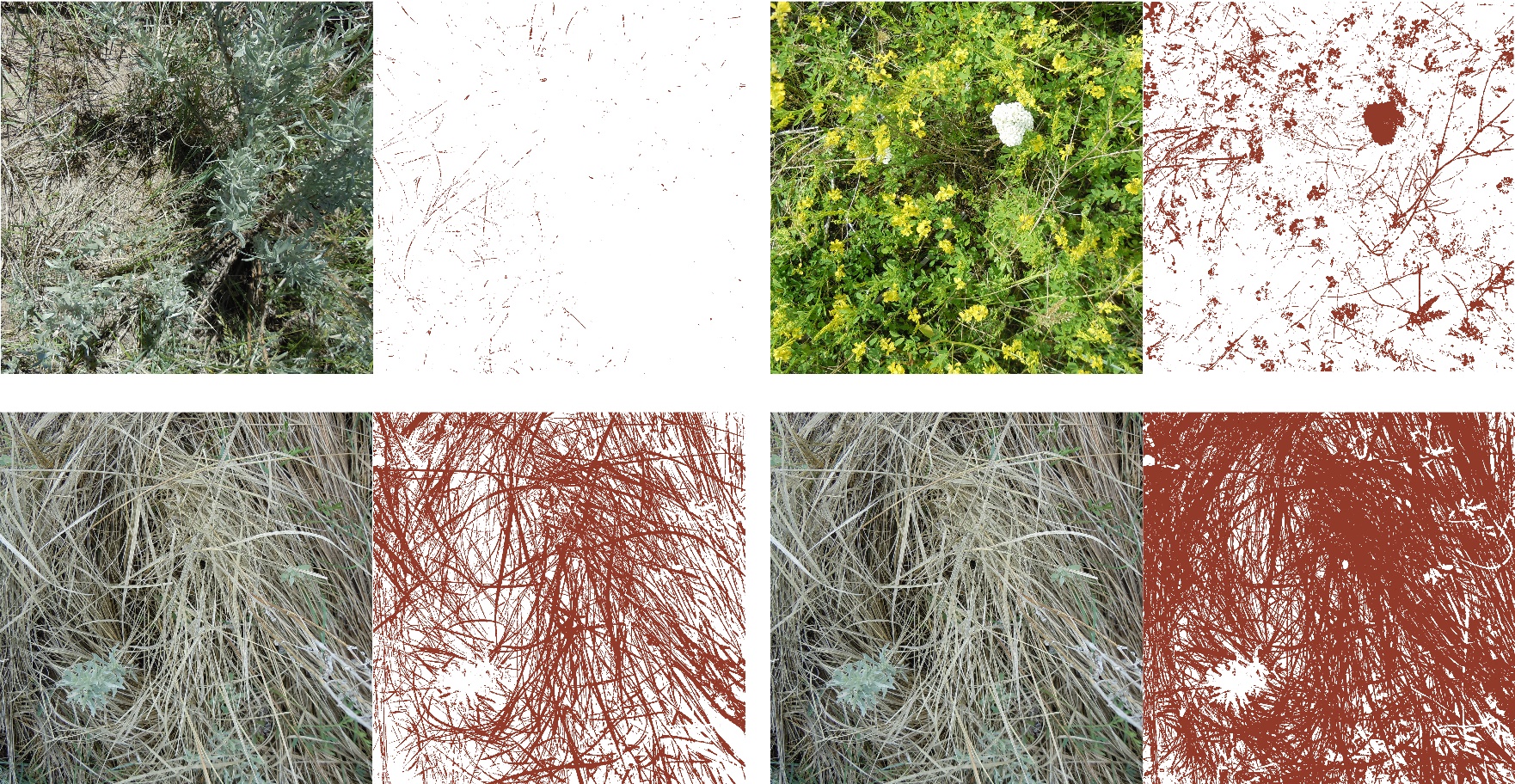


**Figure 9.** Flower effects while extracting standing dead (Constant for SD is 1)

**2. Outputs**

**2.1. Green Vegetation**

It is a raster dataset of extracted green vegetation from the input RGB picture.

**2.2. Standing dead materials**

It is a raster dataset of extracted standing dead vegetation from the input RGB picture.

**2.3. Cover table**

It is a output table including the pixel counts for green vegetation (green_count), pixel counts for standing dead materials (dead_count), total pixel counts for the input RGB picture, standing dead cover in the percentage format (dead_percent) and green cover in the percentage format (green_percent).

**3. Temporary workspace**

It is a temporary workspace used to storage the temporary files.

**4. Process time**

The process time for each RGB picture is about 135s depending on different computers.
