# Supplementary material for: A Semi-Automated Method to Extract Green and Non-Photosynthetic Vegetation Cover from RGB Images in Mixed Grasslands
Source: Sensors (Basel). 2020 Dec 1;20(23):6870. doi: 10.3390/s20236870 (PMC7731437; doi:10.3390/s20236870)
Supplement: Supplementary file 1 [file sensors-20-06870-s001.zip › supplementary S2.pdf]

Supplementary S2

Id	Vegetation community	RGB picture for each quadrat		
1	upland grassland	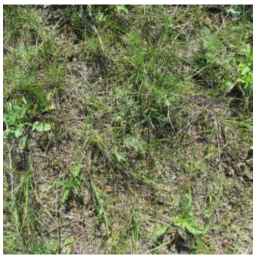	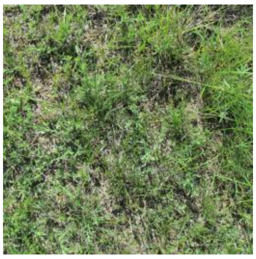	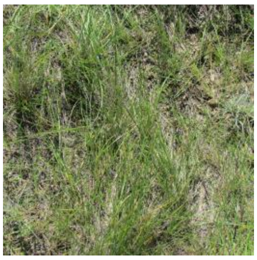
		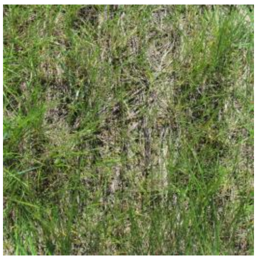	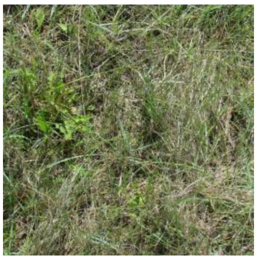	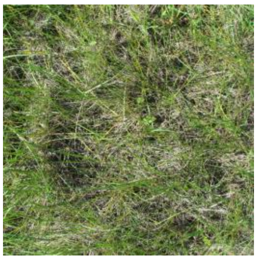
		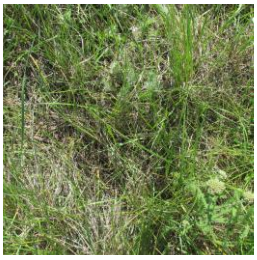	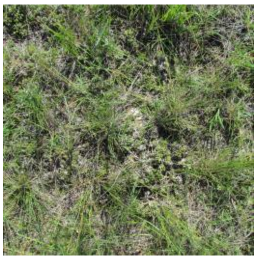	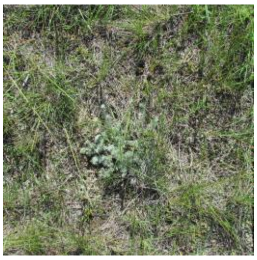
		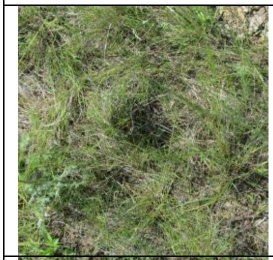	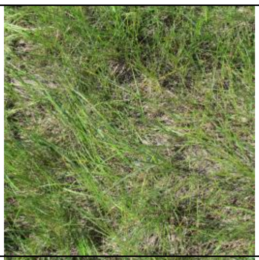	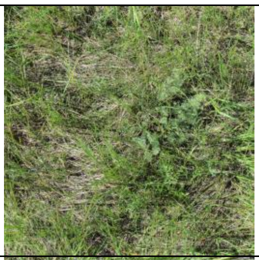
		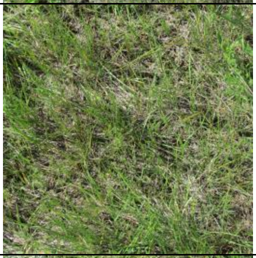	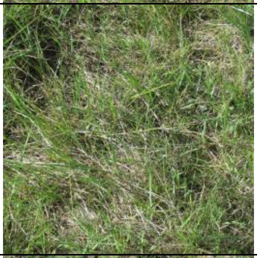	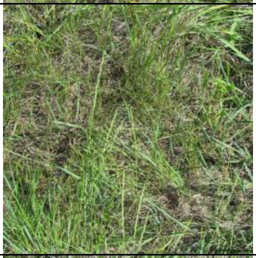
		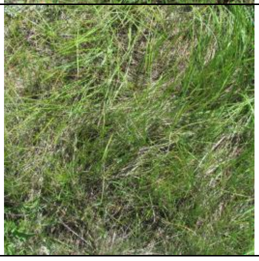	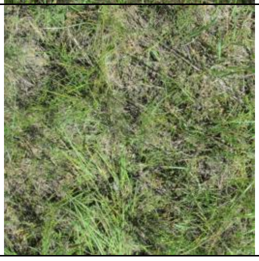	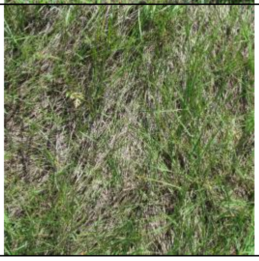

		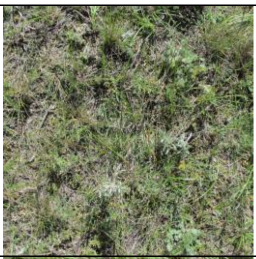		
2	upland grassland	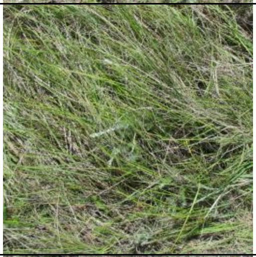	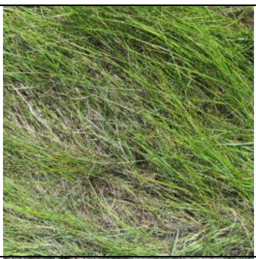	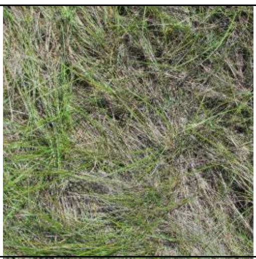
		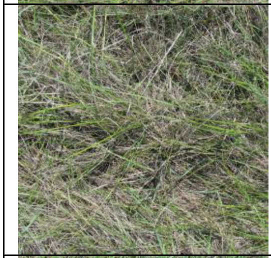	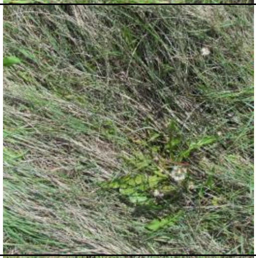	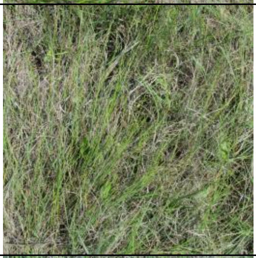
		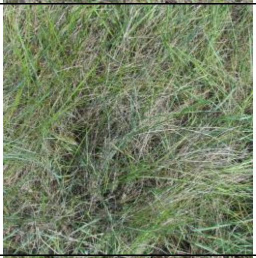	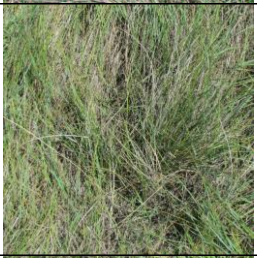	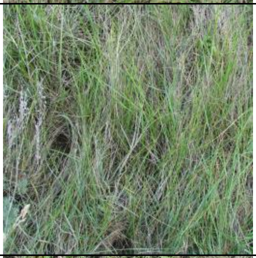
		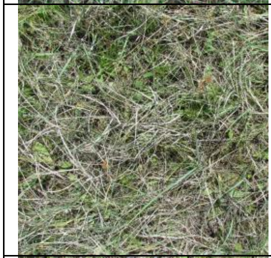	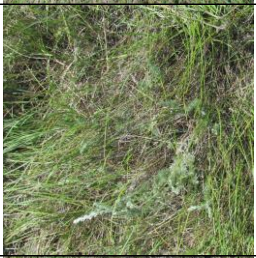	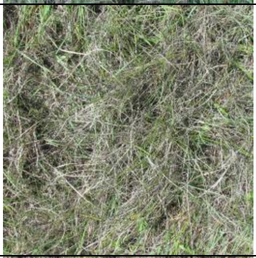
		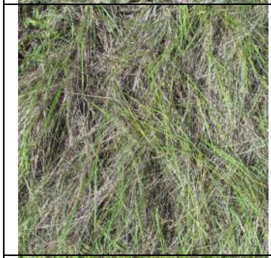	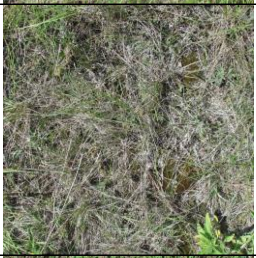	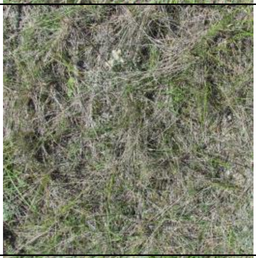
		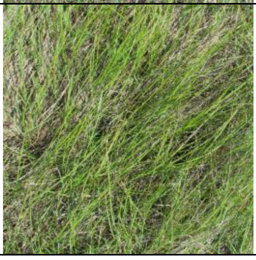	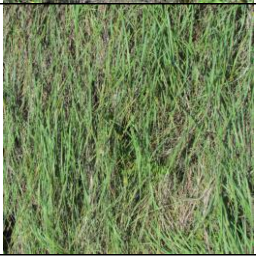	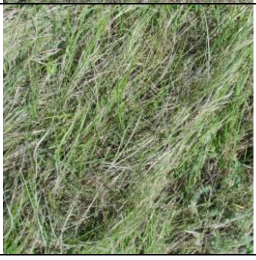

		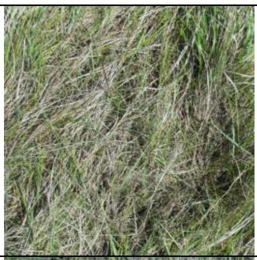	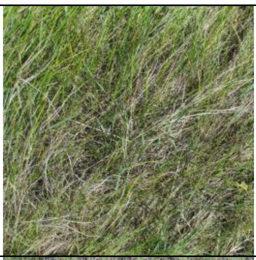	
3	upland grassland	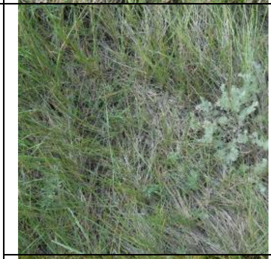	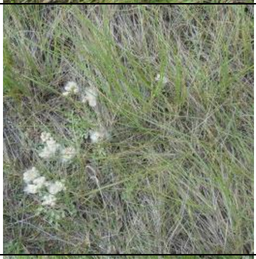	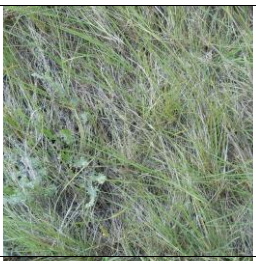
		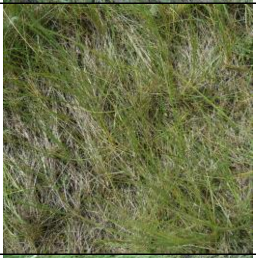	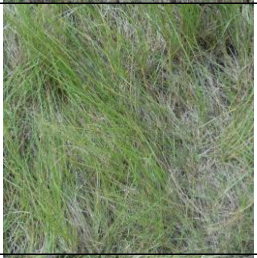	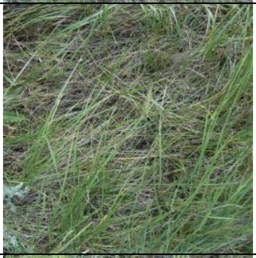
		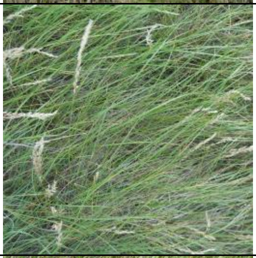	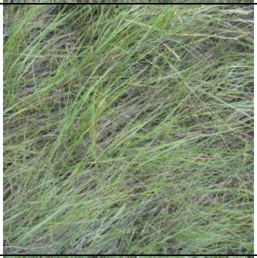	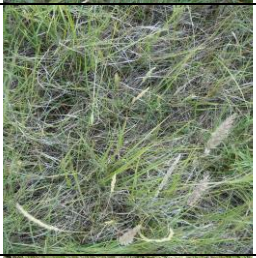
		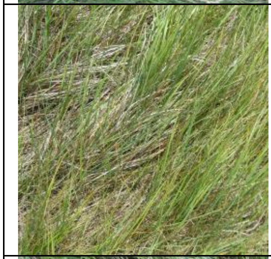	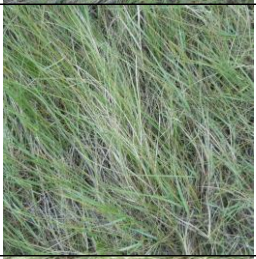	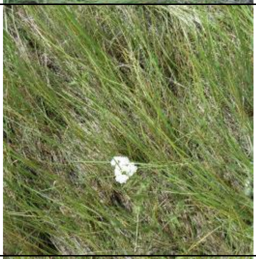
		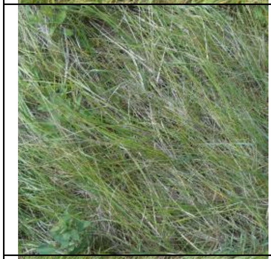	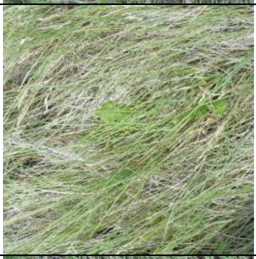	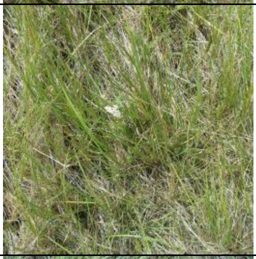
		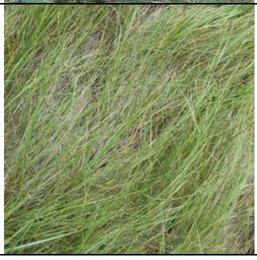	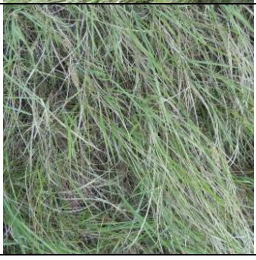	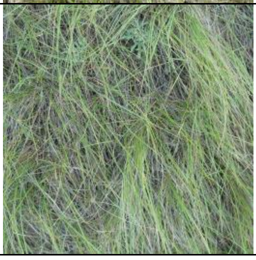

		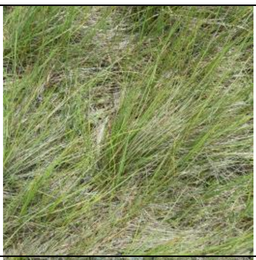	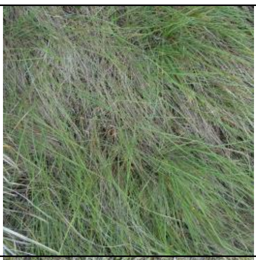	
4	upland grassland	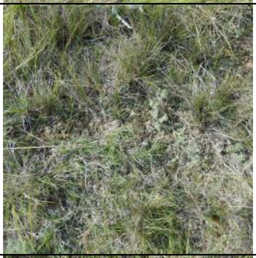	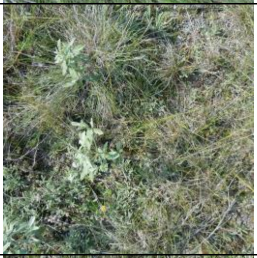	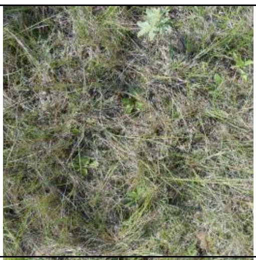
		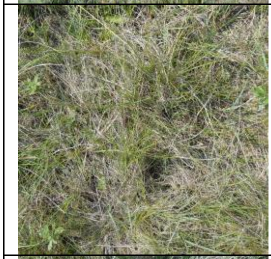	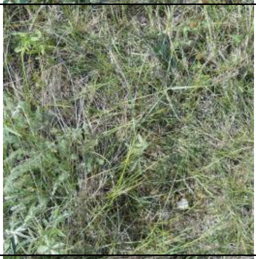	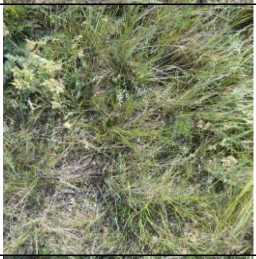
		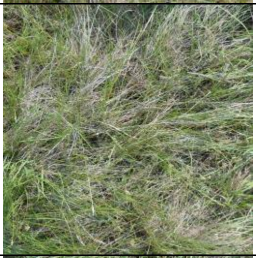	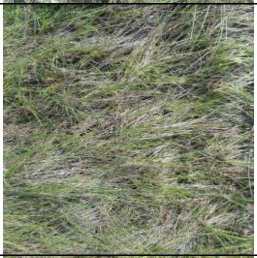	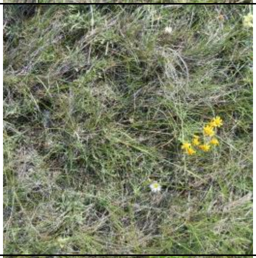
		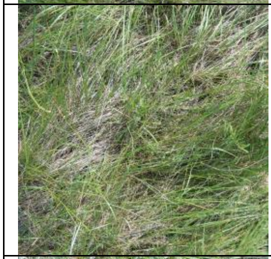	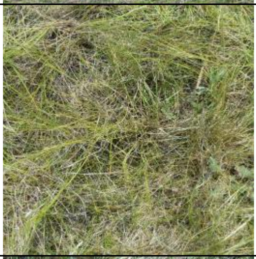	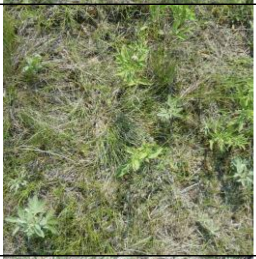
		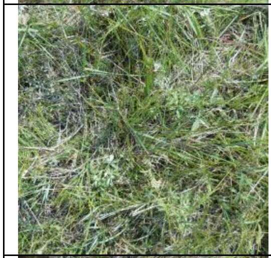	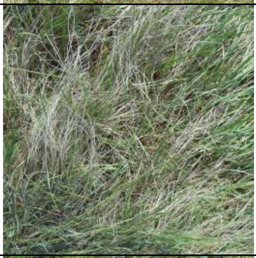	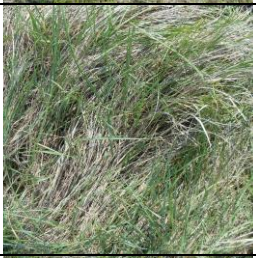
		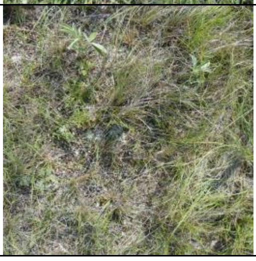	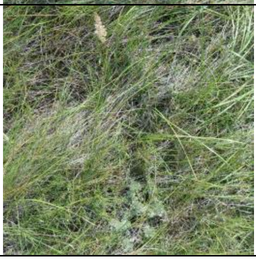	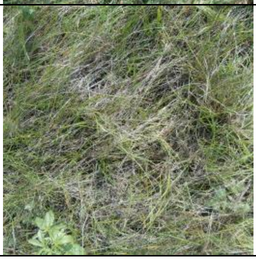

		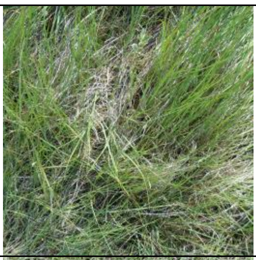	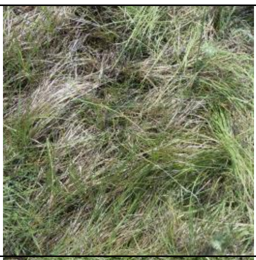	
5	valley grassland	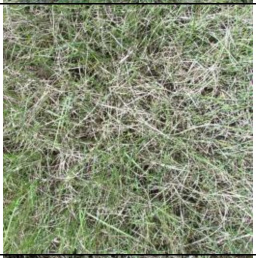	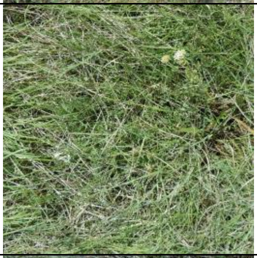	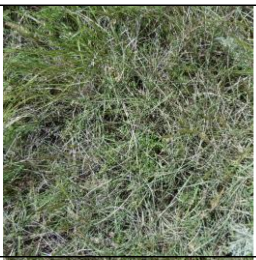
		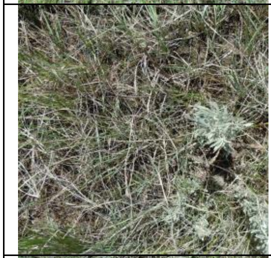	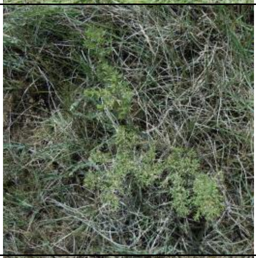	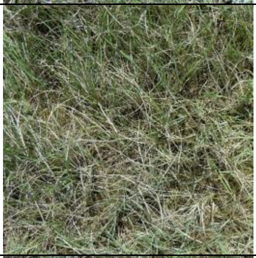
		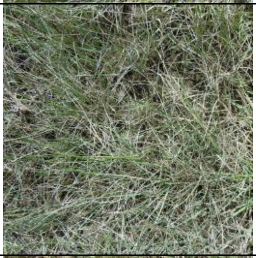	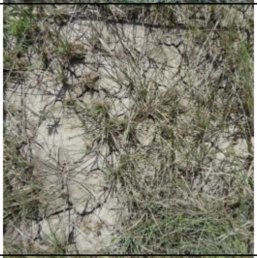	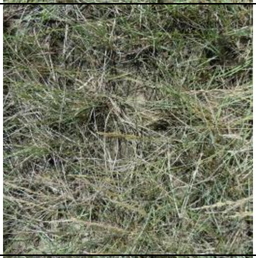
		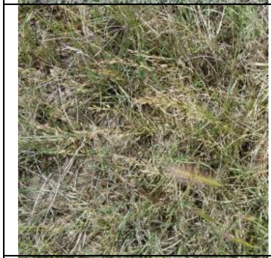	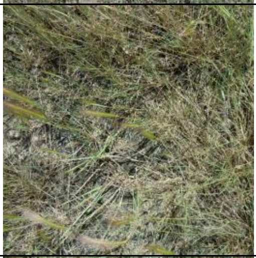	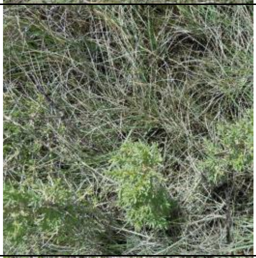
		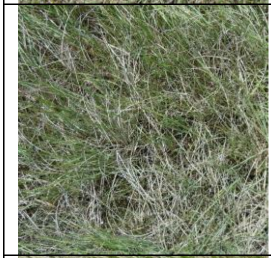	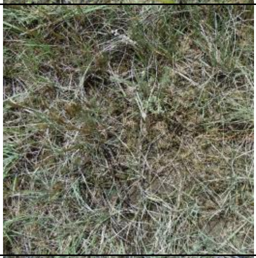	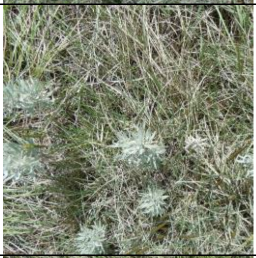
		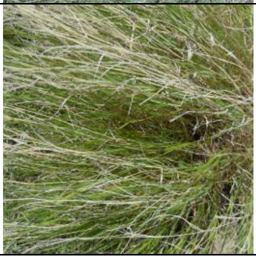	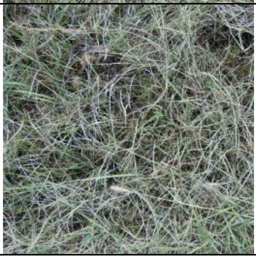	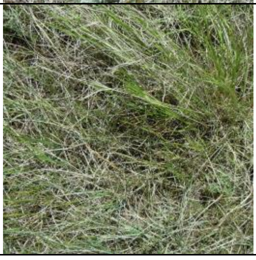

		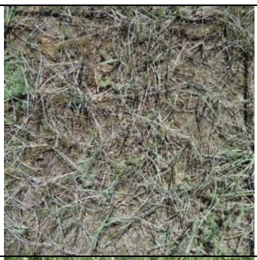	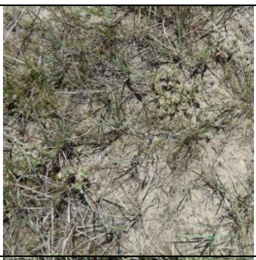	
6	valley grassland	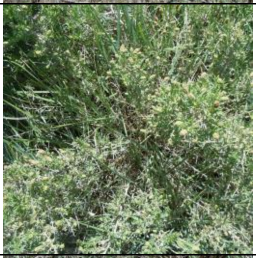	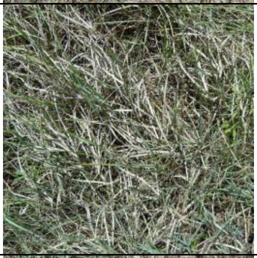	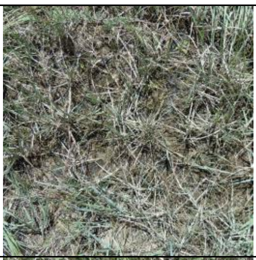
		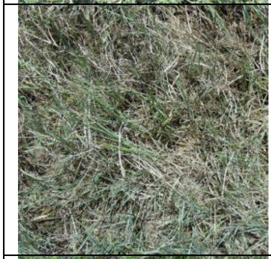	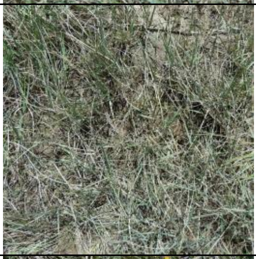	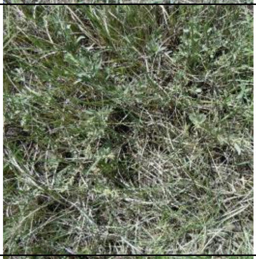
		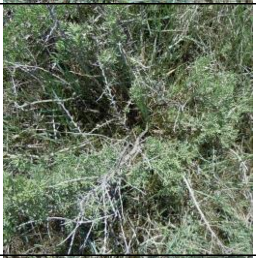	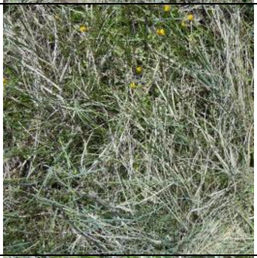	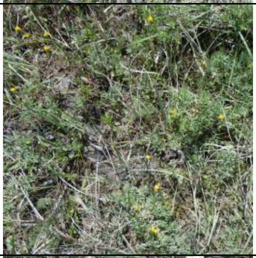
		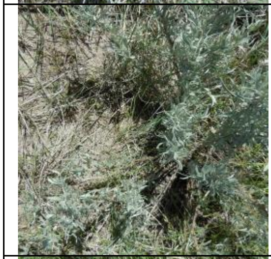	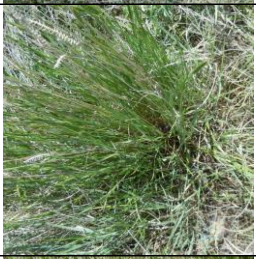	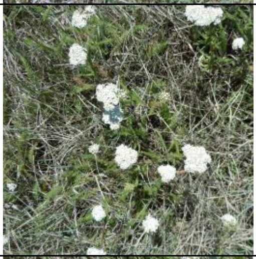
		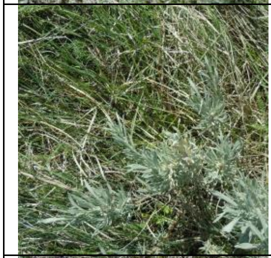	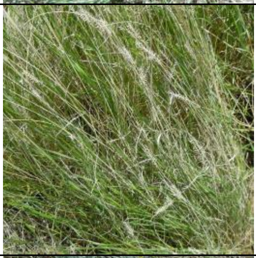	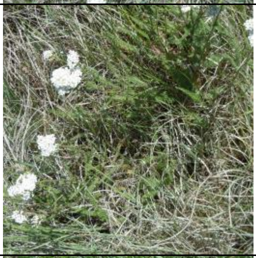
		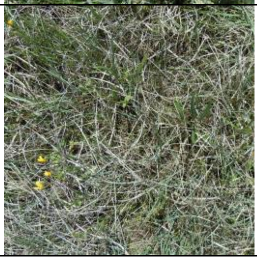	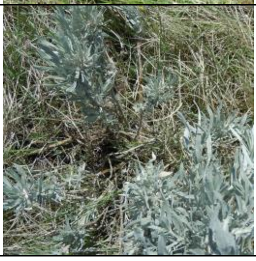	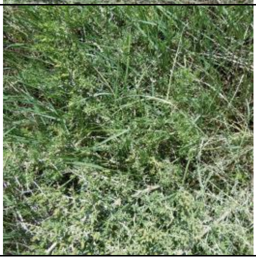

		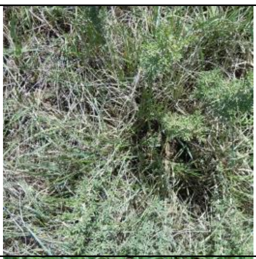	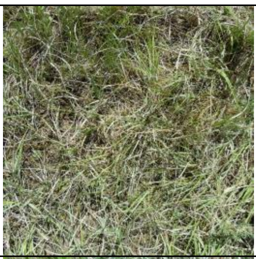	
7	valley grassland	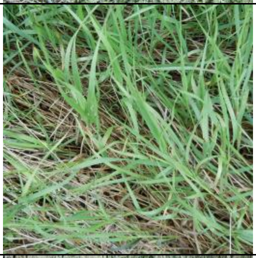	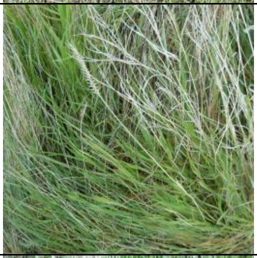	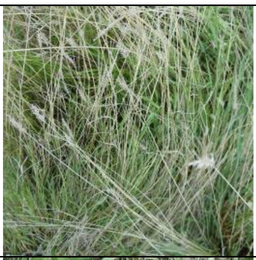
		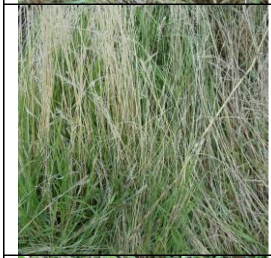	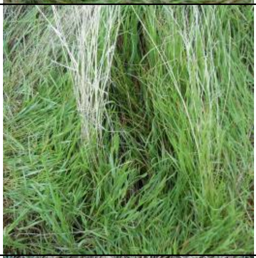	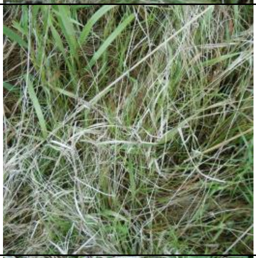
		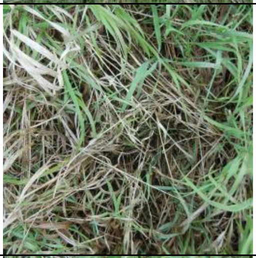	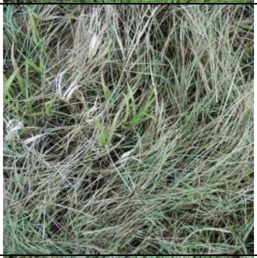	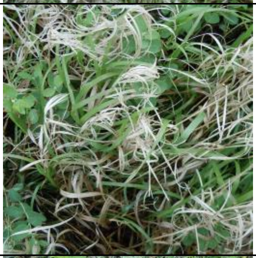
		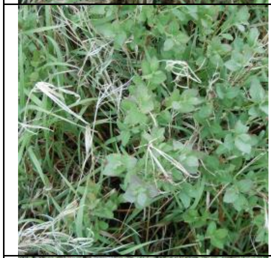	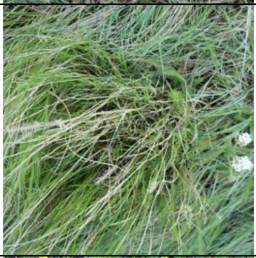	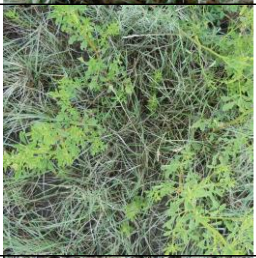
		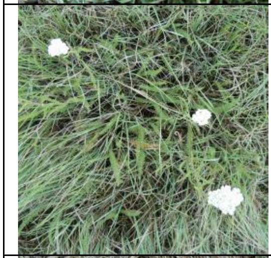	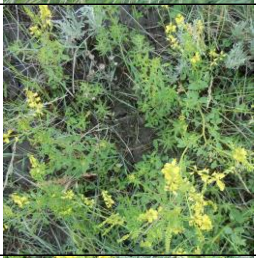	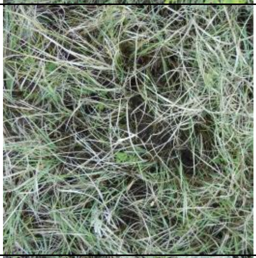
		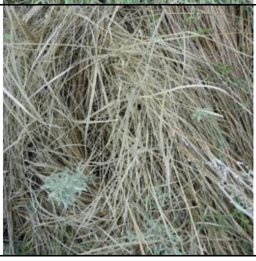	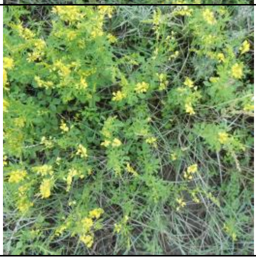	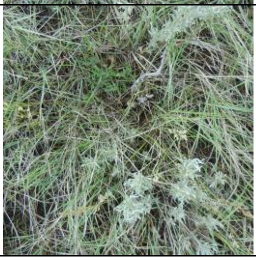

		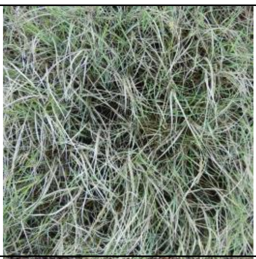	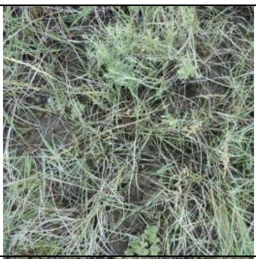	
8	sloped grassland	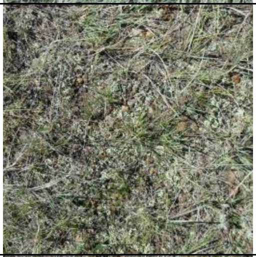	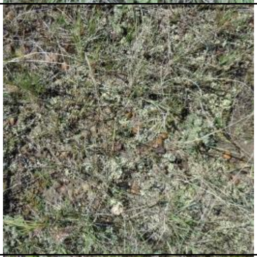	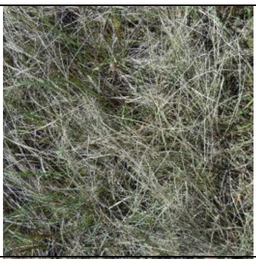
		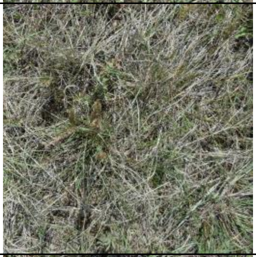	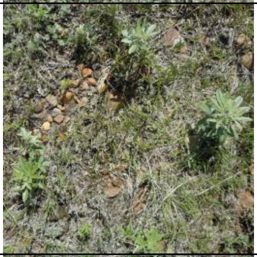	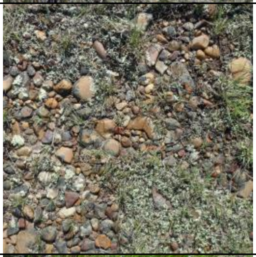
		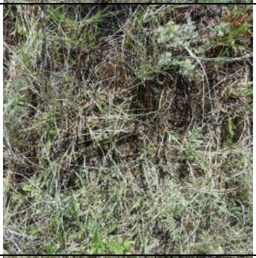	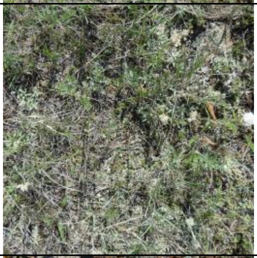	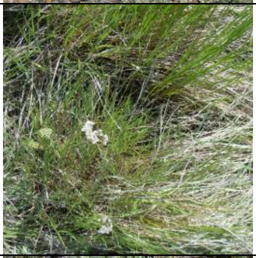
		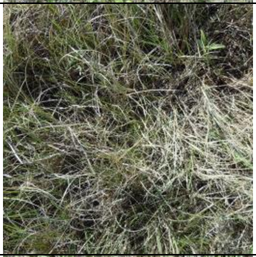	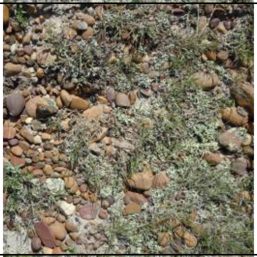	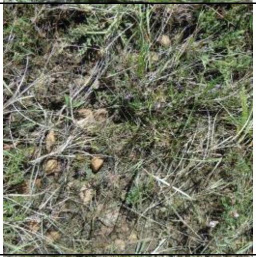
9	sloped grassland	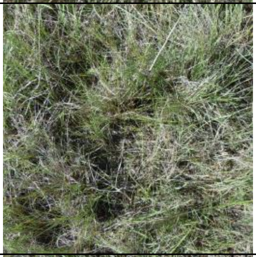	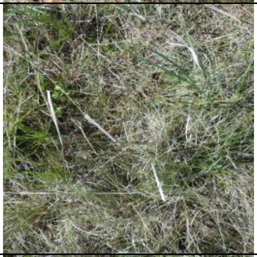	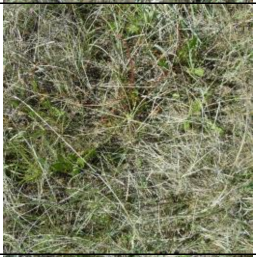
		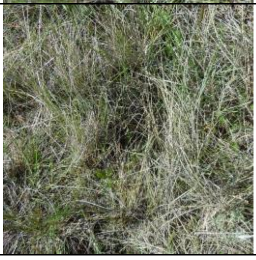	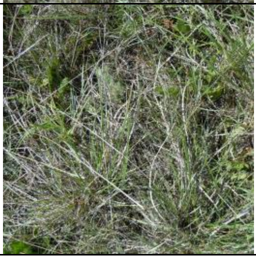	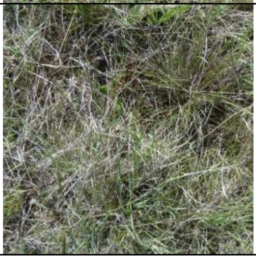

		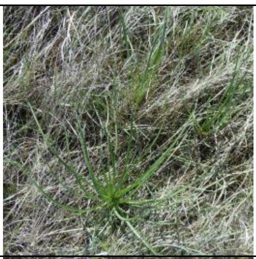	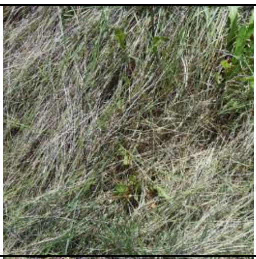	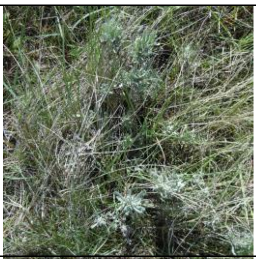
		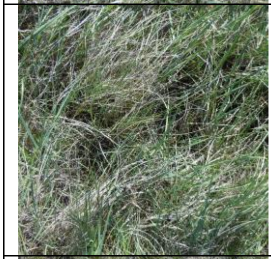	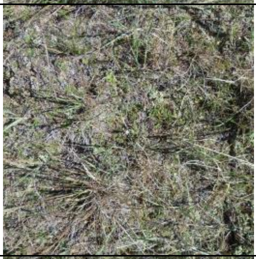	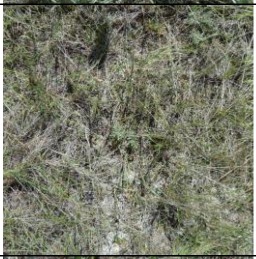
		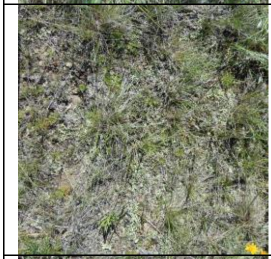	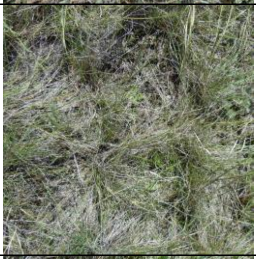	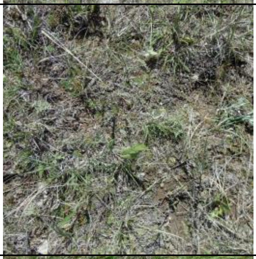
		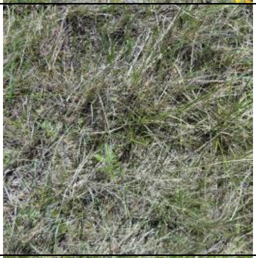	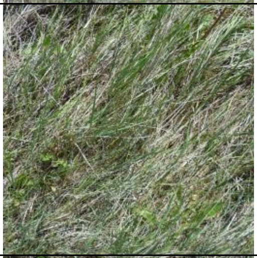	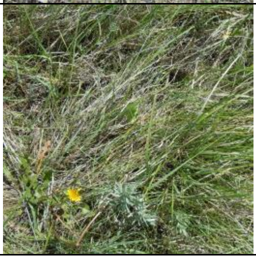
		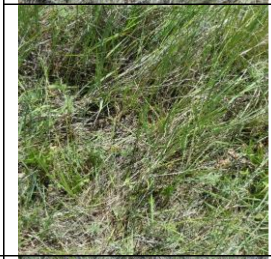	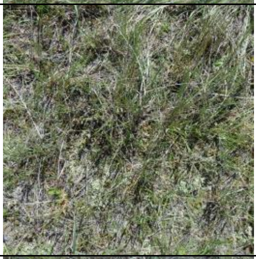	
10	sloped grassland	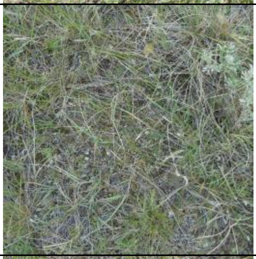	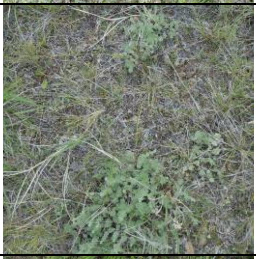	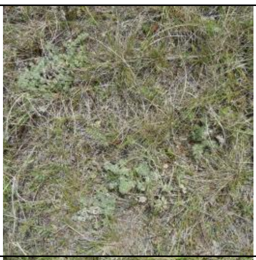
		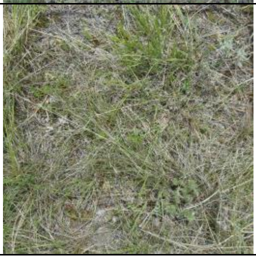	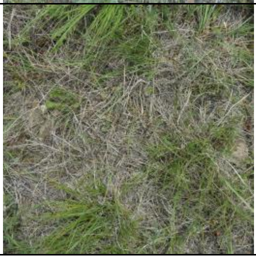	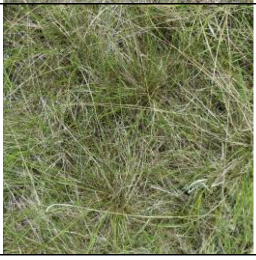

		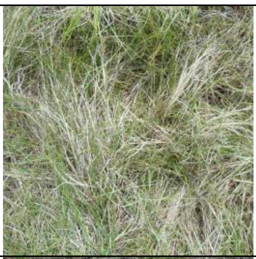	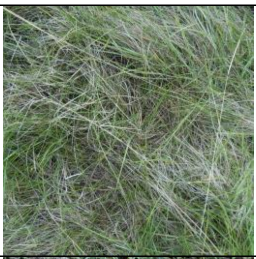	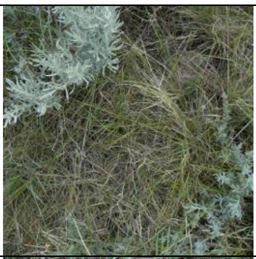
		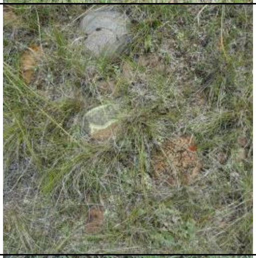	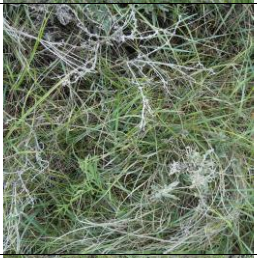	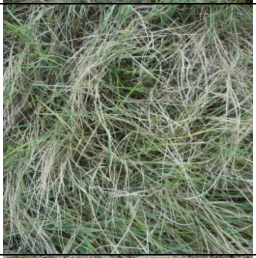
		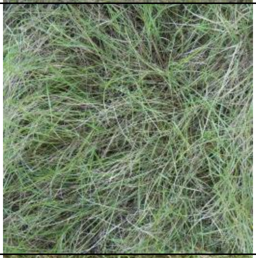	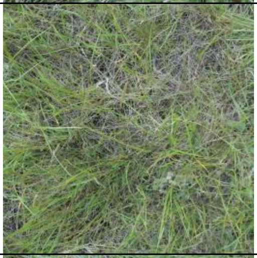	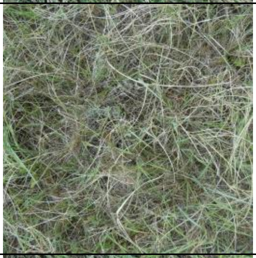
		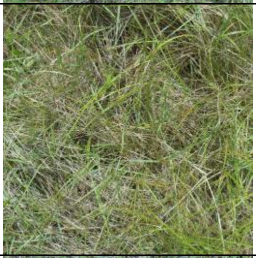	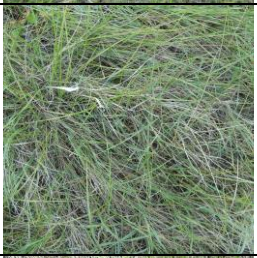	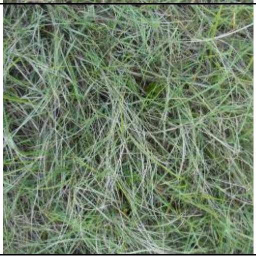
		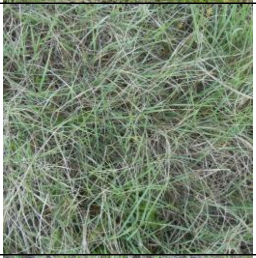	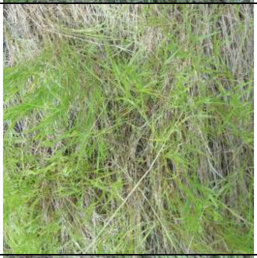	
11	sloped grassland	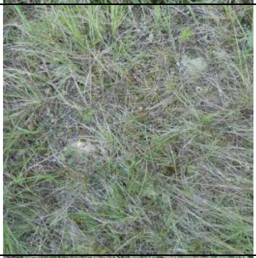	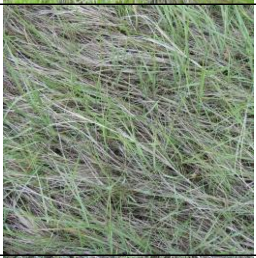	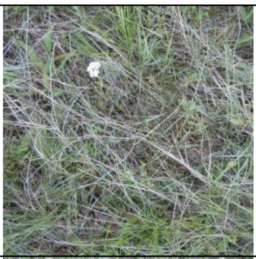
		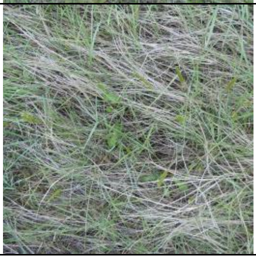	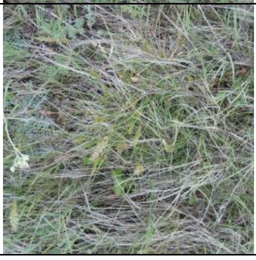	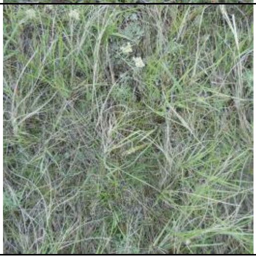

		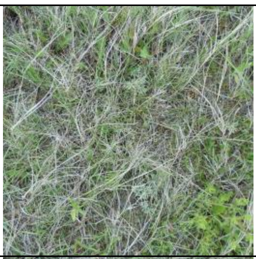	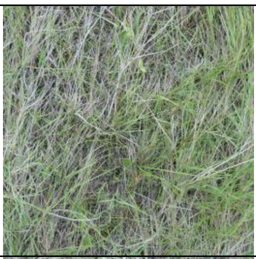	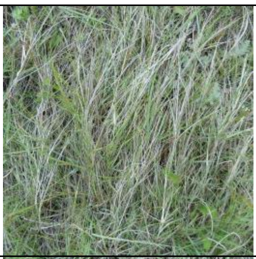
		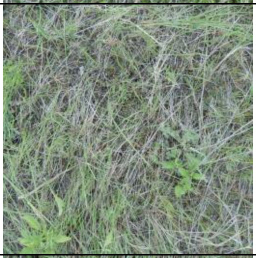	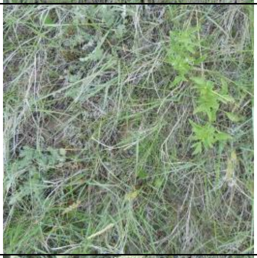	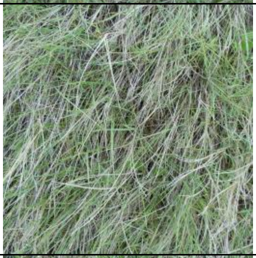
		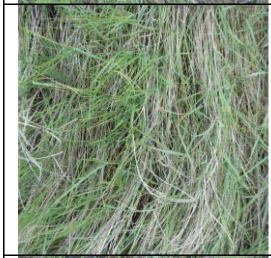	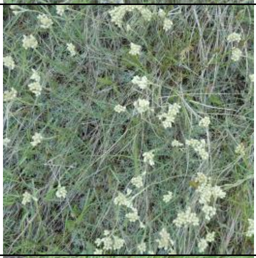	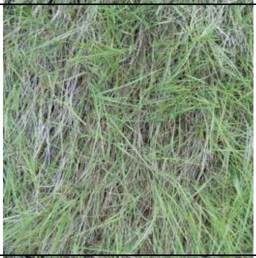
		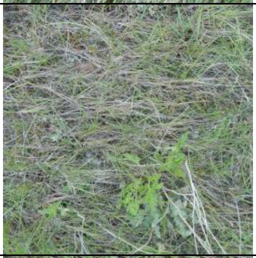	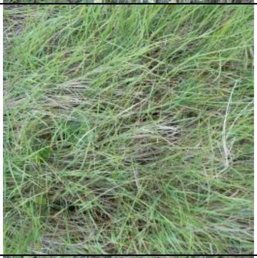	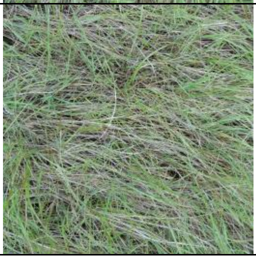
		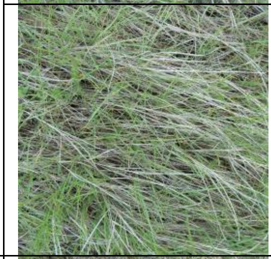	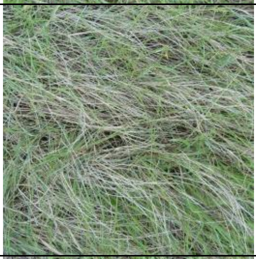	
12	sloped grassland	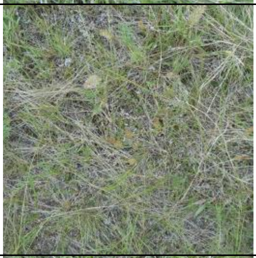	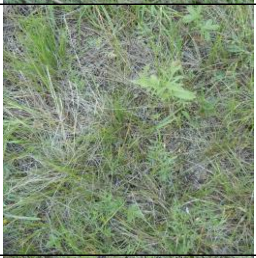	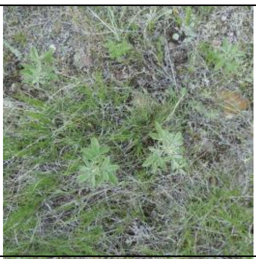
		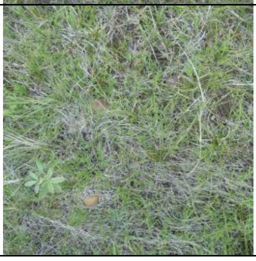	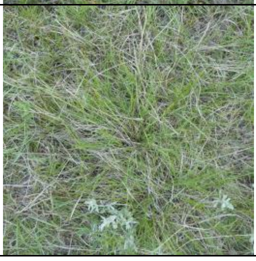	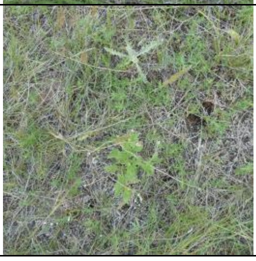

		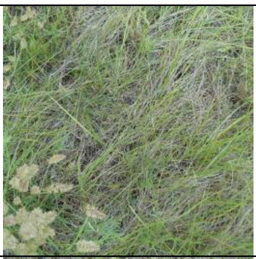	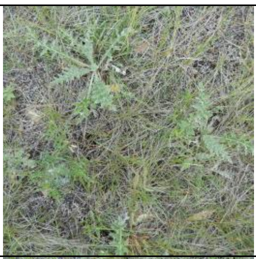	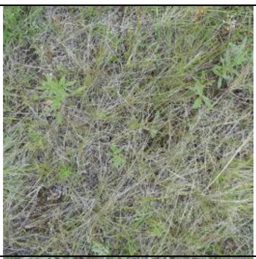
		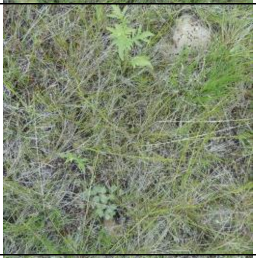	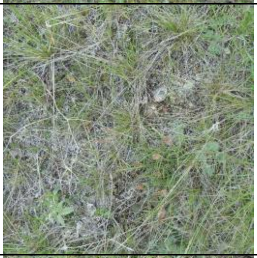	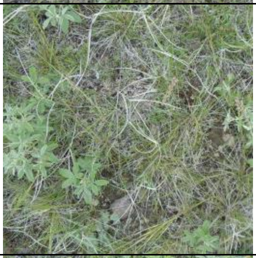
		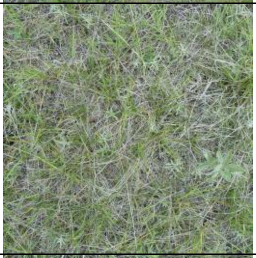	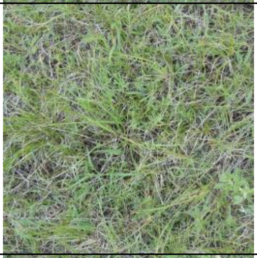	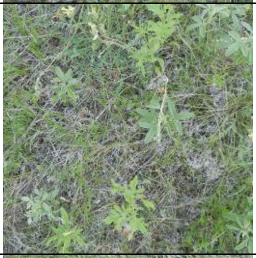
		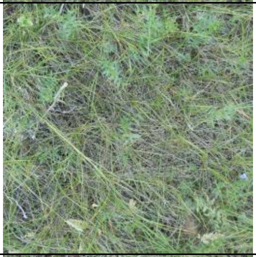	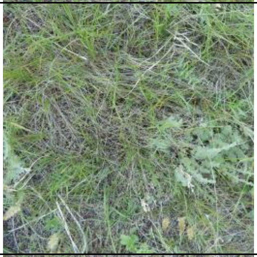	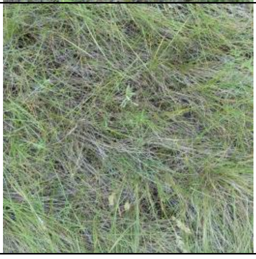
		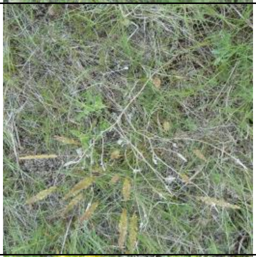	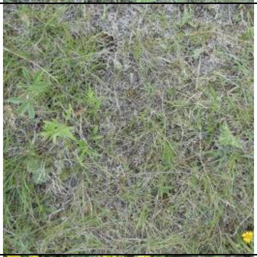	
13	disturbed communities	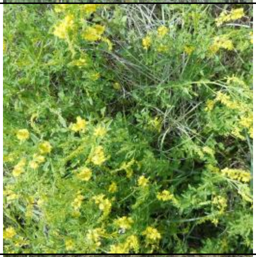	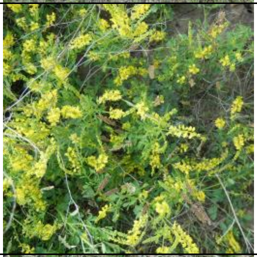	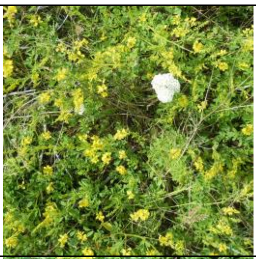
		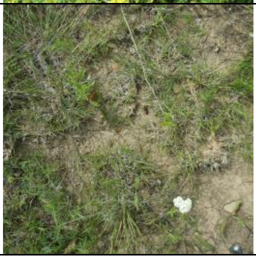	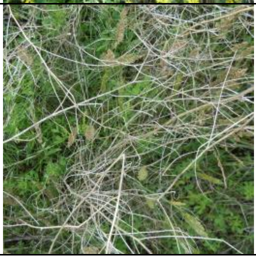	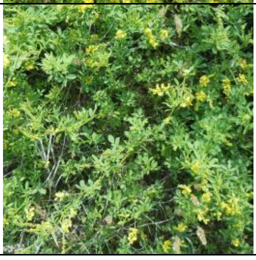

		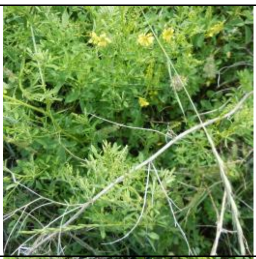	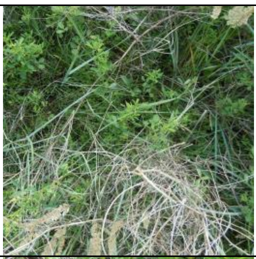	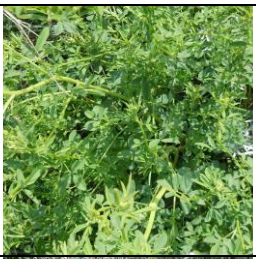
		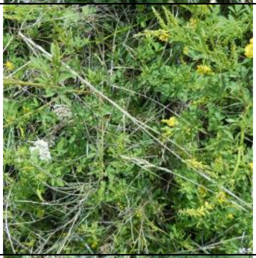	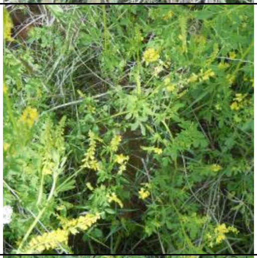	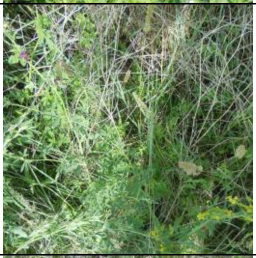
		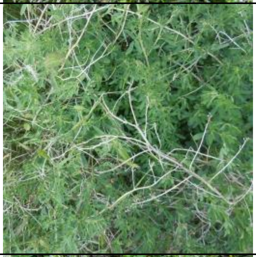	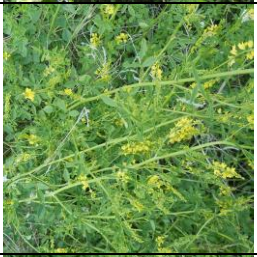	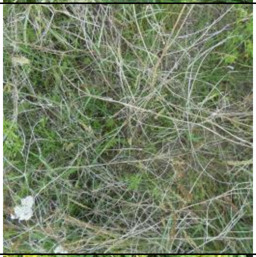
		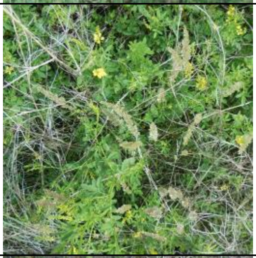	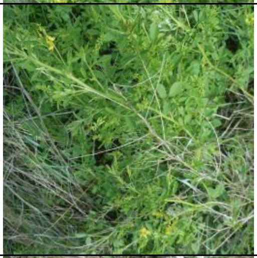	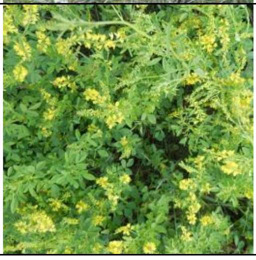
		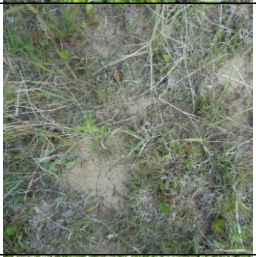	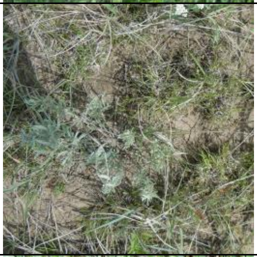	
14	disturbed communities	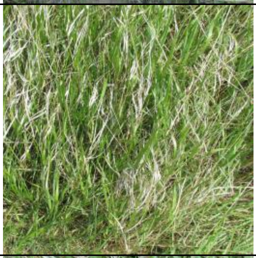	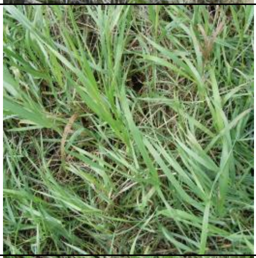	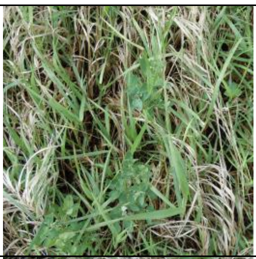
		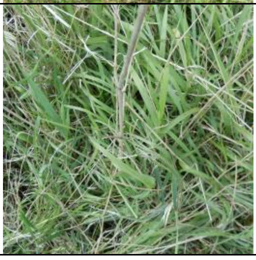	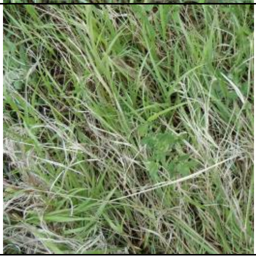	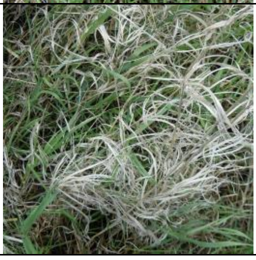

		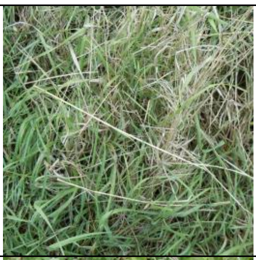	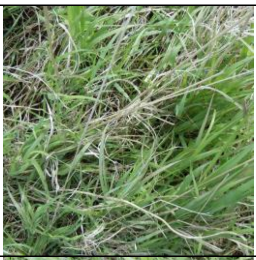	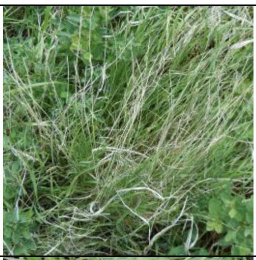
		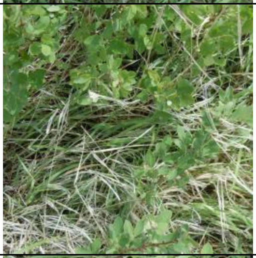	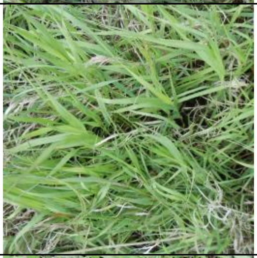	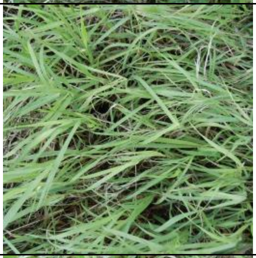
		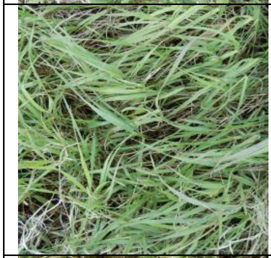	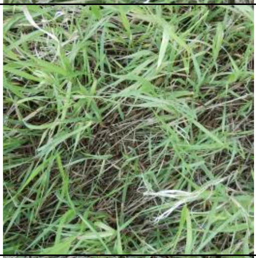	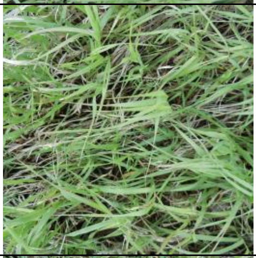
		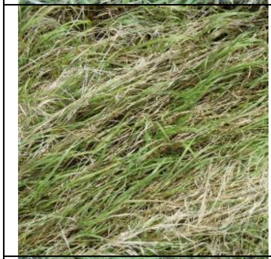	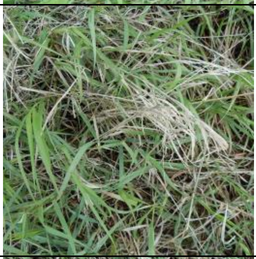	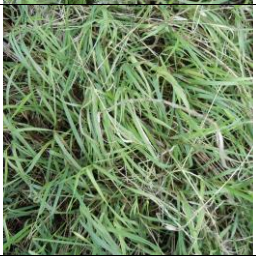
		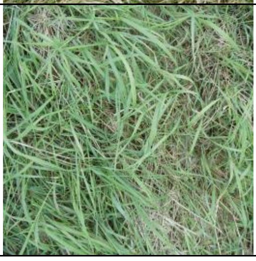	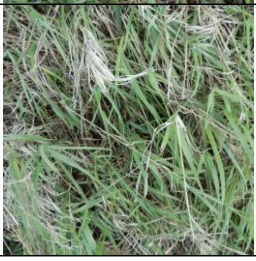	
